# Determination of antimicrobial agents and their transformation products in an agricultural water-soil system modified with manure

**DOI:** 10.1038/s41598-022-22440-5

**Published:** 2022-10-20

**Authors:** Klaudia Stando, Ewa Korzeniewska, Ewa Felis, Monika Harnisz, Martyna Buta-Hubeny, Sylwia Bajkacz

**Affiliations:** 1 Department of Inorganic, Analytical Chemistry and Electrochemistry, Faculty of Chemistry, Silesian University of Technology, B. Krzywoustego 6 Str., 44-100 Gliwice, Poland; 2 Department of Engineering of Water Protection and Environmental Microbiology, Faculty of Geoengineering, University of Warmia and Mazury in Olsztyn, Prawocheńskiego 1 Str., 10-720 Olsztyn, Poland; 3 The Biotechnology Centre, Silesian University of Technology, B. Krzywoustego 8 Str., 44-100 Gliwice, Poland; 4 Environmental Biotechnology Department, Faculty of Power and Environmental Engineering, Silesian University of Technology, Akademicka 2 Str., 44-100 Gliwice, Poland

**Keywords:** Analytical chemistry, Environmental chemistry, Environmental sciences

## Abstract

Manure fertilization is the primary source of veterinary antimicrobials in the water-soil system. The research gap is the fate of antimicrobials after their release into the environment. This study aimed to provide a detailed and multi-faceted examination of fertilized cultivated fields using two types of manure (poultry and bovine) enriched with selected antimicrobials. The research focused on assessing the mobility and stability of antimicrobials in the water-soil system. Additionally, transformation products of antimicrobials in the environment were identified. The extraction (solid-phase extraction and/or solid–liquid extraction) and LC–MS/MS analysis procedures were developed to determine 14 antimicrobials in the soil and pore water samples. Ten out of fourteen antimicrobials were detected in manure-amended soil and pore water samples. The highest concentration in the soil was 109.1 ng g^−1^ (doxycycline), while in pore water, it was 186.6 ng L^−1^ (ciprofloxacin). Sixteen transformation products of antimicrobials were identified in the soil and soil-related pore water. The same transformation products were detected in both soil and soil pore water extracts, with significantly higher signal intensities observed in soil extracts than in water. Transformation products were formed in oxidation, carbonylation, and ring-opening reactions.

## Introduction

Antimicrobial therapy in bovines and pigs is a common practice aimed at increasing the material benefits of meat production^1,2^. Antimicrobial agents (AMs) can be used as feed additives to increase muscle mass and prevent the spread of animal diseases. Due to the fear of the spread of antibiotic-resistant bacterial strains^3,4^ in the European Union, Directive 2001/82/EC and EU Regulation 2019/6 were introduced, prohibiting the use of AMs for preventive purposes.

The mobility of AMs in the environment is particularly dangerous for living organisms such as microorganisms, plants, and aquatic animals. One of the consequences of the presence of AMs residues in the environment may be the spread of antibiotic resistance in various species of bacteria. One hundred ninety-three specific antibiotic resistance genes in bacteria were detected in long-term fertilized soils^5^. The long-term soil fertilization with manure allows AMs to penetrate and accumulate in the environment^6^. The behavior of AMs introduced into an environment depends on its physicochemical properties and environmental factors such as the soil pH, organic matter (including organic carbon), mineral contents, and temperature^7^. AMs are washed away by rainwater, thus contaminating the deeper layers of the soil^8^. AMs sorption coefficients in manure-fertilized soils may be higher than in non-fertilized soils^9^. The complexity of pharmaceuticals' retention and leaching mechanism from the soil has been discussed using selected fluoroquinolones (FQs) and sulfonamides (SAs)^7^. The most important parameters influencing AMs retention were: the soil texture, cation exchange capacity, and pH^7,10,11^. The retention of selected substances in soils is also influenced by organic carbon content, although it is a more complex phenomenon. The correlation between the organic carbon content and the AMs sorption depends on the characteristics of a compound. It should be mentioned that the content of organic carbon in soils is closely correlated with the content of organic matter in the soil. It is assumed that 58% of the soil organic matter (OM) in typical agricultural soils is formed by organic carbon^12^. For example, the sorption of FQs does not depend on the organic matter content, while the sorption of SAs increases upon increasing the organic matter^6^.

The composition of the soil matrix is essential for selecting the analyte extraction method^13^. Table S1 summarizes the validated procedures for extracting pharmaceuticals from solid samples and shows the different recoveries. The most common method is solid–liquid extraction combined with solid phase extraction (SLE-SPE)^14–16^. In SLE the duration, type of solvents, and extraction times are selected empirically to the type of soil sample, which is its major limitation. Soil is a diverse matrix, hence the AMs extraction methods require constant modifications depending on the composition of the environmental sample. There are often problems with the inter-laboratory reproducibility of such procedures for other soil samples. The effectiveness of the extraction of AMs selected from soil samples depends on their composition, organic carbon content, cation exchange capacity, and pH. These parameters are often omitted from publications, therefore extraction methods cannot be treated as universal. LC–MS/MS and LC-HRMS are the most used analytical techniques in environmental samples analysis for their selectivity, high sensitivity, and reproducibility. A literature survey reveals that few analytical methods were reported for quantification of AMs by using LC–MS/MS^14,17–19^, LC-HRMS^20,21^, and less frequently LC-UV^13,22^. LC–MS/MS and LC-HRMS are also used for screening and untargeted analysis, which enables the identification of AMs transformation products in environmental samples^23,24^.

Apart from target AMs, their transformation products (TPs) are also often detected in extracts from soil samples. The most frequent TPs detected in the environmental samples include microorganisms, plant, animal, and human metabolites^25,26^. TPs are also generated during chemical and/or biological wastewater treatment processes and then introduced into the environment with secondary effluents^27^. TPs can also form directly in the environment due to biotic or abiotic factors. The most common abiotic factors transforming AMs are temperature, pH, and UVA/solar radiation^28^. The high complexity of environmental matrices and the low concentrations of AM residues in the environment make the transformation pathways of AMs unclear. A high sensitivity, low detection limit, and the possibility of using various modes of the mass spectrometer make it possible to determine the TPs present in environmental samples^29,30^.

The most commonly observed antimicrobials TPs in environmental samples such as surface water, groundwater, activated sludge and soils are formed via oxidation, hydroxylation, reduction, and acetylation reactions^31,32^. Transformation modeling experiments are often performed at higher concentration ranges (model samples) than in the environment. It should be noted that biotic and abiotic factors act synergistically under natural conditions, and the transformation pathway of AMs in the environment may be different than those under model conditions.

This study aimed to monitor the 14 most commonly used AMs in the agricultural water-soil system. For this purpose, the SLE-SPE-LC–MS/MS method for the isolation and determination of 14 AMs in soil samples was developed and validated. The experiment was conducted in field conditions, using poultry or cattle manure as fertilizer. The field research lasted from late May to early September (4 months), during which the growth and development of vegetables in Poland were more intense (summer-autumn period). The studies determined the stability and the tendency to accumulate AMs pollutants in the soil. As part of the experiment, doxycycline, sulfamethoxazole, tylosin and enrofloxacin were introduced into the soil along with animal manure before the experiment. These four antibiotics are most commonly used to treat poultry and cattle in Poland^33^. Due to the experiment being conducted in environmental conditions, it was decided to expand the list to the 14 most commonly detected AMs^34^ based on the WHO Report on Surveillance of Antibiotic Consumption 2016–2018. First, the isolation and determination methods of 14 AMs (tetracycline (TC), doxycycline (DOX), oxytetracycline (OTC), ciprofloxacin (CIP), enrofloxacin (ENF), levofloxacin (LVF), metronidazole (MET), tylosin (TYL), trimethoprim (TRI), vancomycin (VAN), clarithromycin (CLR), clindamycin (CLD), sulfamethoxazole (SMX), and sulfadiazine (SFD)) from soil samples fertilized with animal manure were developed and validated. LC–MS/MS was used to monitor the AMs content in soil samples. An additional aim of the study was to perform screening and non-targeted analysis to identify the TPs of selected AMs under environmental conditions.

## Materials and methods

### Standards, chemicals, and materials

Full details of the standards, chemicals, and materials used in this work are available in Supplementary Material S1. The standard stock solutions of 11 selected AMs with a concentration of 1.0 mg mL^−1^ were diluted in methanol. The three remaining AMs were dissolved in: 0.1% formic acid in methanol (CIP), acetone (SFD), and methanol:water (1:1; v/v, VAN). Working solutions of the AMs were prepared in the range of 1.0–600.0 ng mL^−1^.

### Sample collection and characterization

The study was conducted at the University of Warmia and Mazury Experimental Station, located in Tomaszkowo, Poland (53° 42′ 35.6″ N 20° 26′ 05.0″ E). In the field conditions, 12 experimental plots were prepared in five variants: (1) soil plots supplemented with poultry manure and AMs (PMPA; 3 plots); (2) soil plots supplemented with bovine manure and AMs (BMPA: 3 plots); (3) poultry manure soil plots (PMP; 2 plots); (4) bovine manure soil plots (BMP; 2 plots); (5) control plots without supplementation (manure or AMs) (CP; 2 plots). For PMPA and BMPA, selected AMs (doxycycline—DOX, enrofloxacin—ENF, sulfamethoxazole—SMX, and tylosin—TYL) were added at a concentration of 50 mg of each AMs per 1 kg of manure. Next, manure samples were homogenized by stirring. The area of a single plot was 4 m^2^. Soil and pore water samples were collected four times: before enrichment (day 0) and after enrichment on days 1, 14, and 133. The soil was fertilized with enriched manure to simulate the natural introduction of AMs into the environment. No AMs were applied to the remaining plots (PMP, BMP, CP). The amount of manure used for fertilization was 1.5 kg m^2^ for poultry and 4 kg m^−2^ for bovine.

The soil was sampled from a depth of 0–20 cm with a soil sampling stick in 100 mL sterile plastic containers. Five independent samples were taken from each plot according to the envelope method. The sampling stick was sterilized with ethanol before each sampling. After collection, samples were immediately delivered to the laboratory, protected from light during transport, and stored in a refrigerated container. After delivery to the laboratory, the samples were freeze-dried and homogenized. Composite soil samples were prepared by combining all samples from one plot type. The fresh weight and dry weight of soils were recorded. Freeze-dried composite samples were stored in the dark at −20 °C until analysis.

Soil pore water was collected at 30, 60, 90, and 120 cm depths. The sampling apparatus was automated and consisted of four samplers, filters placed at the bottom of the samplers, and a pressure chamber (Geomor Technik, Poland). The water samples were transferred to sterile plastic containers and transported to the laboratory in a darkened, refrigerated container. After delivery to the laboratory, the total volume of each sample (parameter dependent on atmospheric conditions) and basic physicochemical properties (pH, conductivity) were measured. Samples were stored at −20 °C until analysis (a maximum of 3 days from delivery to the laboratory).

### Sample preparation

To obtain the highest possible recoveries for the studied soil samples enriched with animal manure, the procedure was divided into three separate steps: (1) solid–liquid extraction (SLE) optimization; (2) solid-phase extraction (SPE) optimization; (3) evaporation, dissolving, and filtration. All experiments were carried out in three replicates, and each sample was analyzed twice using liquid chromatography coupled with a tandem mass spectrometry (LC–MS/MS). Complete information about the solvent selection, extraction time, use of ultrasound-assisted extraction (UAE), selection of SPE conditions, and sample preparation before analysis are available in Supplementary Material S2.

Extraction solvents were selected based on Table S1. In the optimization of SLE, solvent mixtures in various volume ratios, the effect of shaking time and intensity, the effect of ultrasound, and the stability of analytes in different solvents were examined.

The next step was SPE. Different SPE cartridges were evaluated: OASIS HLB (500 mg, 6 mL), OASIS WAX (60 mg, 3 mL), and Bakerbond C18 (200 mg, 3 mL) cartridges. Next, sample pH, and elution solvent were considered as following: (A) sample pH = 3; cartridges and eluents: HLB (12 mL methanol) + WAX (5 mL methanol), (B) sample pH = 4; cartridges and eluents: HLB (12 mL methanol) + WAX (5 mL methanol), (C) sample pH = 4; cartridges and eluents: HLB (6 mL methanol + 6 mL 0.1% acetic acid in methanol) + WAX (3 mL methanol + 3 mL 0.1% acetic acid in methanol), (D) sample pH = 3; cartridges and eluents: HLB (6 mL methanol + 6 mL 0.1% ammonia in methanol) + WAX (3 mL methanol + 3 mL 0.1% ammonia in methanol), (E) sample pH = 3; cartridges and eluents: HLB (6 mL methanol + 4 mL 0.1% acetic acid in methanol) + WAX (3 mL methanol + 2 mL 0.1% acetic acid in methanol).

#### Soil sample extraction procedure

Before extraction, soil samples were homogenized and freeze-dried. The soil sample (model sample) was enriched with a mixture of AMs (250 ng g^−1^, dry weight) and left for 24 h. To extract AMs from soil samples, SLE and SPE were used. During the development of the SLE step, mixed solutions of inorganic solvents (citrate buffer, McIlvaine buffer, or sodium hydroxide) with an organic solvent (methanol, acetonitrile, or acetone) were selected to test the extraction efficiency of AMs from enriched soil. Different experimental conditions were tested to optimize the SLE of the target AMs from spiked soils. Details of the selection of SLE and SPE parameters during individual steps of the procedure are placed in the supplementary material. Finally, two variants of the SLE-SPE procedure were used to extract AMs from soil samples. Procedure I was for TYL, TRI, MET, SFD, CLD, and CLR extraction, while procedure II was for TC, DOX, OTC, CLD, VAN, CIP, LVF, and ENF.

The natural soil (PMPA, BMPA) was enriched with 4 (DOX, ENF, SMX, TYL) of the 14 AMs ("Sample preparation" section). The study was extended using ten additional analytes to assess whether AMs exist in the environment and how their content changes during the study.

**Procedure I**

Freeze-dried soil (2 g) samples were weighed, and then 10 mL of McIlvaine buffer (pH = 4):methanol (1:1; v/v) was added and shaken for 30 min. The sample was mixed for 30 min at 900 rpm and centrifuged for 10 min at 8000 rpm. The supernatant was then decanted into a 500 mL glass bottle. The soil was re-extracted with 10 mL of McIlvaine buffer (pH = 4): acetonitrile (1:1; v/v) for 30 min. The supernatants were combined, diluted with 200 mL of distilled water, and adjusted to pH = 3 with formic acid.

**Procedure II**

Freeze-dried solids (2 g) were weighed, and then 10 mL of McIlvaine buffer (pH = 4): methanol (1:1; v/v) was added. The sample was mixed for 30 min at 900 rpm and centrifuged for 10 min at 8000 rpm. The supernatant was then decanted into a 500 mL glass bottle. Then, extraction with 0.2 M sodium hydroxide solution:acetone (1:1; v/v) was performed by shaking the sample for 20 min and centrifuging for 10 min. The supernatant was adjusted to pH 3 with formic acid. Both extracts were combined and diluted to 250 mL with distilled water. The pH of the sample was measured and adjusted to pH = 3 if necessary.

**Figure 1:**
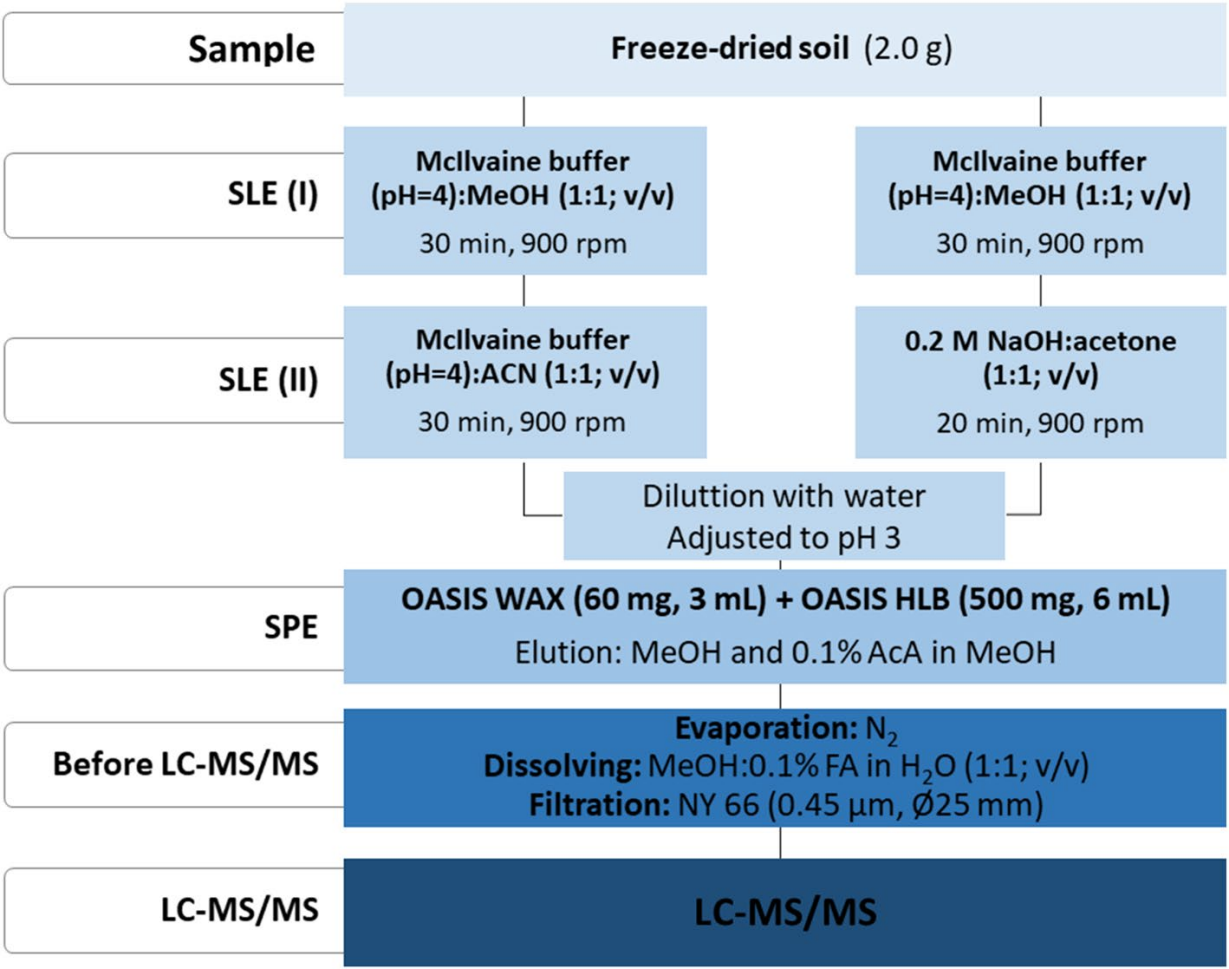
Schemat of the developed extraction procedure.

The SPE conditions were the same for both procedures. The Baker® SPE-12 G system (vacuum manifold for 12 cartridges, Witko, Poland) equipped with a pump (LABOPORT™ PowerDry™ Vacuum Pump, KNF Neuberger, USA) was used. For SPE optimization, triplicate blank soil extract (without analyte, after SLE) was spiked with the standard mixed solution at 500 ng L^−1^. Cartridges were placed in tandem to simultaneously remove negatively-charged humic material (WAX; 60 mg, 3 mL) and retain the AMs (HLB; 500 mg, 6 mL). The WAX cartridge was placed on the top of the HLB, and both columns were conditioned using 6 mL methanol followed by 6 mL 0.1 M hydrochloric acid and 6 mL water. The diluted extract was loaded into the tandem cartridges at approximately 3 mL min^−1^. The cartridges were dried under vacuum for 20 min. Finally, the analytes were eluted separately from the WAX and HLB columns by 3 mL methanol and 2 mL 0.1% acetic acid in methanol for the WAX cartridge and 6 mL methanol and 4 mL 0.1% acetic acid in methanol for the HLB cartridge. The eluents were combined and evaporated to dryness under a stream of nitrogen. The dry extracts were then reconstituted with 1 mL 0.1% formic acid:methanol (1:1; v/v). The extract was then filtered through a nylon syringe filter (0.45 µm, Ø25 mm; PureLand, CHEMLAND, Stargard, Poland) and transferred to a 1 mL glass vial. The extract was analyzed using LC–MS/MS. To summarize, the newly developed step-by-step SLE-SPE procedure included (I) SLE Procedure 1, (II) SLE Procedure 2, (III) tandem SPE, and (IV) evaporation + dissolving + filtration (Fig. 1).

#### Soil pore water sample extraction procedure

The SPE procedure used to prepare liquid samples has been described in a previous report^35^. Briefly, the pore water samples were filtered through an MN-GF-1 glass fiber filter (MACHERY-NAGEL, Dueren, Germany) and adjusted to pH = 3 with sulfuric acid (96%). An OASIS HLB cartridge (500 mg, 6 mL) was conditioned with 6 mL methanol and 6 mL 0.1 M hydrochloric acid, and then the samples were loaded at a flow rate of 5 mL min^−1^. The cartridges were vacuum-dried for 15 min and then eluted with 20 mL of methanol. The samples were evaporated and dissolved in 1.0 mL of methanol before LC–MS/MS analysis.

### Instrumental and analytical conditions

Chromatographic separation of 14 selected AMs was performed with a Dionex HPLC system (Dionex Corporation, Sunnyvale, CA, USA) equipped with a Zorbax SB-C3 column (150 mm × 3.0 mm, 5 μm). Dionex Chromeleon TM 6.8 software was used for analysis control. The mobile phase of the chromatographic system consisted of acetonitrile (A) and 0.1% formic acid in water (B). Separation was performed at 30 °C at a flow rate of 1.0 mL min^−1^. Initially, the proportion of solvent A in the mobile phase was 10%, which was constant for two minutes. After the second minute, the proportion of solvent A in the mobile phase increased linearly during 3 min to 35% (from 2.0 min to 5.0 min). From 5.0 min to 7.0 min, the content of solvent A increased linearly, and its maximum content was 85%. The column was equilibrated for 3 min until the next injection. The total analysis time was 10 min. An injection volume of 2 µL was employed for all samples.

The HPLC system was interfaced to an AB Sciex Q-Trap® 4000 mass spectrometer (Applied Biosystems/MDS SCIEX, Foster City, CA, USA) with an electrospray ionization source operated in positive-ion mode (ESI +). Data acquisition was performed in multiple reaction monitoring (MRM) mode, where two precursor-product ions were chosen for each AM. Analyst 1.4 software was used for instrument control, data acquisition, and processing. The source temperature was maintained at 500 °C, ion spray voltage (IS) was 4000 V, curtain gas (CUR) was at 10 psi, collision gas (CAD) was medium, ion source gas 1 (GS1) was 60 psi, and ion source gas 2 (GS2) was 50 psi. The MRM transition and MS/MS parameters are given in Table S2.

### Method validation

The developed LC–MS/MS method for analyzing AMs in soil was validated. The analytical method was evaluated by determining selectivity, sensitivity, matrix effect, recovery, accuracy, and precision. The description is provided in Supplementary Material S3.

### Determination of the antimicrobial agents and identification of their TPs in soil and soil pore water samples

Selected AMs were determined in the soil and pore water from Warmian-Masurian Voivodeship. Soil material and pore water were sampled from 12 experimental plots exposed to atmospheric factors. The soil material was collected at a maximum depth of 20 cm, while the pore water was collected at four depths: 30, 60, 90, and 120 cm. After delivering the water samples to the laboratory, they were filtered through a glass fiber filter, and then the volume and pH were measured. Soil samples were weighed and then freeze-dried. After lyophilization, the samples were reweighed and then homogenized. Then, the analytes were extracted by SLE-SPE according to the procedure described in "Instrumental and analytical conditions" section.

The targeted analysis was performed using the LC–MS/MS method in MRM mode. Non-targeted analysis was also performed to detect the TPs of selected AMs, and modes of high-performance ion trap (IT) were applied. The TPs were detected using information-dependent acquisition (IDA) with enhanced product ion (EPI) and enhanced mass scan (EMS). Next, the degradation products resulting from biotic and abiotic factors were confirmed based on information available in the literature. The operating parameters of the mass spectrometer were the same as those used for the targeted analysis.

## Results and discussion

### Step by step to the effective extraction procedure: development of the SLE-SPE conditions for extraction of AMs from soil samples

The solid extraction procedures described in the literature (Table S1) are based on a combined SLE and concentration step and purification using SPE; however, using the conditions described in the literature gave recoveries of < 50% for each analyte. Soil matrices are a diverse group of environmental samples, and there is no single extraction procedure that can ensure a high recovery of analytes in samples with extremely various physicochemical parameters. Only a few publications provide the physicochemical characteristics of tested soil samples along with the extraction procedure^36–39^. The lack of data on the nature of soil sample testing significantly reduces the reproducibility of the described procedures in other laboratory conditions. As shown in Table S1, there is no one-size-fits-all procedure for extracting the same groups of analytes from the soil. Most often, solutions buffered in a mixture with an organic solvent are used for SLE. However, they differ in the extraction duration, the number of repetitions, and additional steps, such as ultrasound assistance or rotary evaporation.

**Figure 2:**
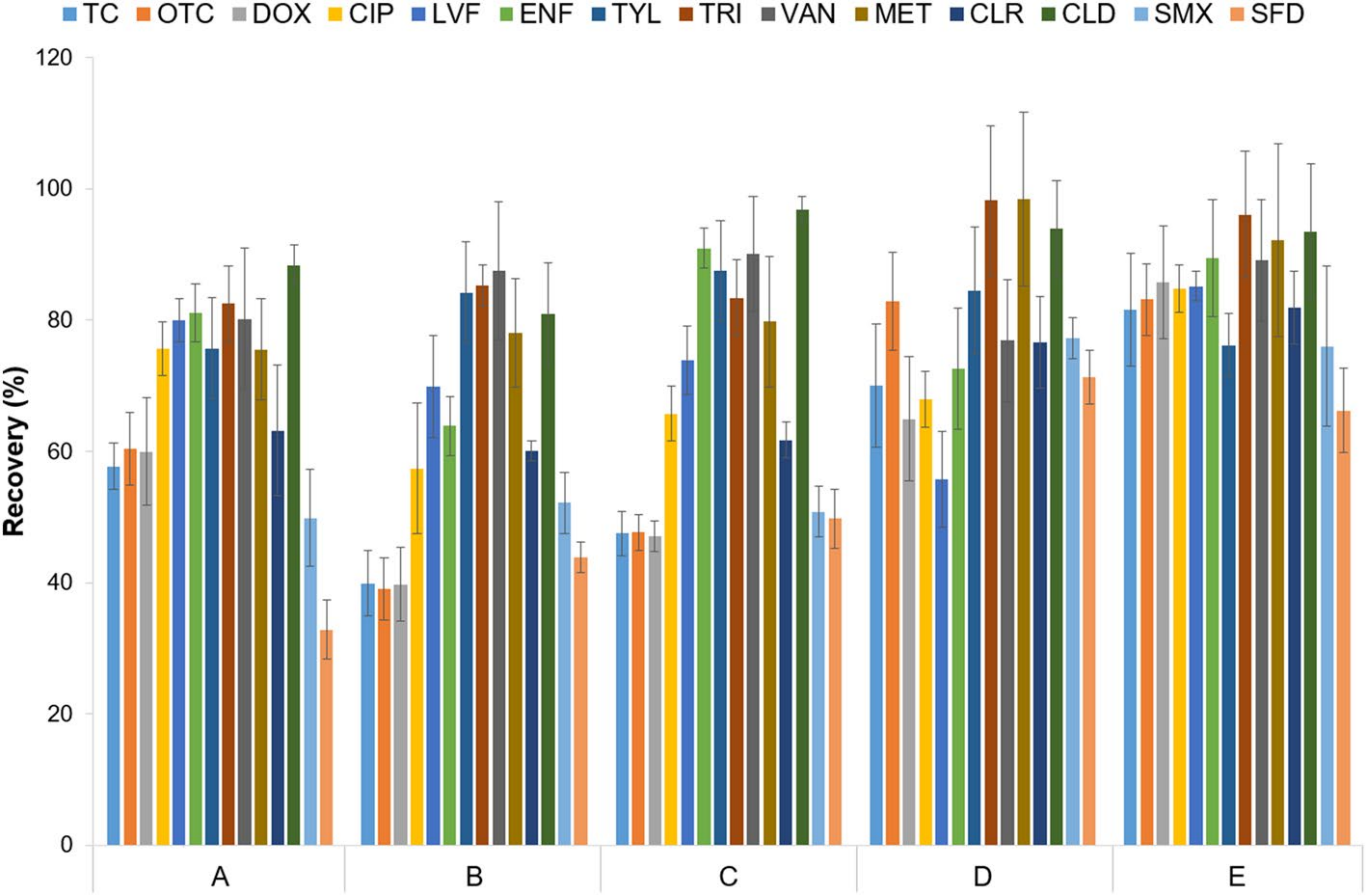
Comparison of SPE conditions (**A**) sample pH = 3; cartridges and eluents: HLB (12 mL methanol) + WAX (5 mL methanol); (**B**) sample pH = 4; cartridges and eluents: HLB (12 mL methanol) + WAX (5 mL methanol); (**C**) sample pH = 4; cartridges and eluents: HLB (6 mL methanol + 6 mL 0.1% acetic acid in methanol) + WAX (3 mL methanol + 3 mL 0.1% acetic acid in methanol); (**D**) sample pH = 3; cartridges and eluents: HLB (6 mL methanol + 6 mL 0.1% ammonia in methanol) + WAX (3 mL methanol + 3 mL 0.1% ammonia in methanol); (**E**) sample pH = 3; cartridges and eluents: HLB (6 mL methanol + 4 mL 0.1% acetic acid in methanol) + WAX (3 mL methanol + 2 mL 0.1% acetic acid in methanol).

The efficiency of a single SLE step was assessed, and the results are summarized in Table 1. Figure 2 shows the analyte recoveries after SPE using various extraction parameters (described in "Soil sample extraction procedure" section ). A full description of the development SLE-SPE procedure was described in Supplementary Material S4.

**Table 1:** Effect of the solvent in SLE procedure applied for extraction antimicrobials from soil.

Antimicrobials recovery (%) (SD (%))														
Solvent	TC	OTC	DOX	CIP	LVF	ENF	TYL	TRI	VAN	MET	CLR	CLD	SMX	SFD
Citrate buffer (pH = 4):ACN (1:1; v/v)	31.7 (2.1)	26.3 (1.7)	30.6 (5.4)	10.8 (2.0)	15.8 (2.1)	12.8 (3.7)	103.2 (7.8)	98.0 (8.5)	58.1 (4.9)	100.5 (8.8)	89.6 (7.9)	110.1 (12.2)	88.1 (9.0)	104.0 (3.9)
Citrate buffer (pH = 4):MeOH (1:1; v/v)	27.7 (2.2)	31.3 (2.1)	33.1 (1.5)	5.2 (1.5)	4.7 (0.5)	4.7 (0.7)	86.2 (6.7)	78.1 (4.4)	65.1 (5.9)	83.0 (17.6)	79.6 (4.6)	91.7 (12.9)	78.4 (1.9)	97.8 (3.5)
0.1% FA in MeOH	–	–	–	1.4 (0.1)	11.7 (1.3)	2.1 (0.5)	88.5 (10.2)	89.0 (2.3)	–	85.4 (8.7)	86.7 (1.6)	90.0 (14.0)	68.0 (4.5)	76.3 (6.3)
MeOH:AcEt (1:1; v/v)	–	–	–	0.0 (0.0)	0.0 (0.0)	0.0 (0.0)	97.5 (10.9)	84.9 (5.3)	–	103.9 (11.5)	67.4 (2.8)	98.4 (11.1)	72.0 (6.1)	69.3 (5.1)
0.2 M NaOH in H_2_O	–	–	–	39.1 (2.1)	29.9 (1.2)	53.4 (9.6)	33.9 (0.5)	94.0 (4.8)	117.4 (8.6)	80.5 (8.9)	61.3 (4.2)	79.8 (6.4)	69.1 (4.2)	63.8 (2.2)
2% NH_3_ in MeOH	–	–	–	5.1 (0.6)	5.1 (0.7)	4.0 (0.7)	61.1 (3.6)	93.7 (6.2)	3.5 (0.1)	53.6 (6.3)	48.9 (3.2)	68.2 (8.8)	46.7 (5.8)	50.8 (4.3)
ACN:0.1% FA in H_2_O (1:1; v/v)	–	–	–	3.6 (0.6)	6.2 (0.7)	5.5 (0.3)	104.6 (4.6)	91.1 (12.1)	66.2 (6.4)	77.5 (8.6)	83.0 (3.8)	72.3 (10.5)	84.0 (2.0)	63.3 (0.6)
Citrate buffer (pH = 2):ACN (1:1; v/v)	15.5 (1.6)	14.1 (1.8)	1.8 (0.28)	9.4 (1.2)	13.1 (1.6)	14.7 (2.6)	68.1 (4.1)	90.1 (4.6)	86.6 (1.6)	83.1 (13.3)	54.1 (4.3)	87.5 (14.1)	54.6 (2.3)	96.0 (3.7)
McIlvaine buffer (pH = 4):MeOH (1:1; v/v)	48.7 (4.7)	48.1 (5.7)	44.7 (5.5)	3.9 (0.2)	5.5 (0.7)	2.6 (0.2)	71.2 (5.8)	63.5 (7.5)	75.2 (8.3)	85.6 (6.4)	90.3 (7.2)	90.2 (6.8)	96.1 (7.1)	84.6 (12.2)
McIlvaine buffer (pH = 4):ACN (1:1; v/v)	86.7 (9.5)	79.7 (7.3)	83.7 (7.7)	22.9 (2.4)	25.4 (4.7)	29.3 (2.7)	79.1 (2.5)	85.3 (4.9)	71.0 (10.5)	74.9 (3.9)	89.6 (7.9)	82.9 (11.0)	84.1 (8.2)	81.7 (4.9)
MeOH:ACN:0.1 M EDTA:McIlvaine buffer (pH = 4) (30:20:25:25; v/v/v/v)	57.9 (7.9)	59.2 (6.2)	93.9 (8.7)	14.2 (1.7)	26.8 (3.3)	15.9 (1.2)	81.7 (3.1)	81.8 (3.5)	96.7 (9.3)	83.3 (3.1)	86.9 (6.6)	85.9 (14.5)	95.5 (55.7)	88.2 (2.7)
ACN:McIlvaine buffer (pH = 4):0.1 M EDTA (2:1:1; v/v/v)	70.6 (6.0)	67.4 (8.3)	88.3 (4.4)	15.8 (0.8)	16.5 (4.5)	20.9 (1.2)	79.4 (1.0)	90.9 (6.1)	92.9 (9.5)	55.8 (3.6)	80.4 (10.6)	88.2 (10.9)	89.0 (3.7)	59.0 (2.98)
0.2 M NaOH in H_2_O:MeOH (1:1; v/v)	2.9 (0.2)	2.9 (0.2)	–	75.3 (5.0)	54.8 (12.2)	85.6 (5.1)	40.2 (5.4)	98.5 (5.0)	79.5 (9.2)	62.2 (3.4)	81.7 (2.2)	71.08 (6.4)	89.8 (14.8)	115.0 (16.9)
0.2 M NaOH in H_2_O:ACN (1:1; v/v)	5.4 (0.7)	5.8 (1.1)	6.3 (0.8)	79.5 (3.9)	96.8 (4.8)	86.9 (4.0)	70.0 (2.3)	104.9 (14.6)	96.3 (1.7)	59.6 (14.0)	80.1 (14.9)	90.5 (5.7)	94.4 (11.6)	82.6 (6.6)
0.2 M NaOH in H_2_O:acetone (1:1; v/v)	0.7 (0.1)	1.4 (0.1)	–	85.4 (10.9)	107.3 (17.0)	86.5 (7.9)	2.34 (0.3)	60.8 (9.4)	66.9 (7.3)	64.3 (5.9)	81.8 (9.6)	72.9 (2.1)	88.2 (11.7)	88.0 (11.9)

ACN—acetonitrile, FA—formic acid, AcEt—ethyl acetate, “–”—no data (no peak in the chromatogram).

### Method validation

The LC–MS/MS method's applied conditions were validated and the results were presented in Table 2 and Supplementary Material S5. The recovery of analytes from soils in optimized procedures was: 60–95% (procedure 1) and 70–96% (procedure 2) in HQL.

**Table 2:** The analytical method parameters and extraction recovery of antimicrobials in soil samples at three concentrations (*n* = 6).

Analyte	Linear range (ng g^−1^)	R^2,a^	LOD^b^ (ng g^−1^)	LOQ^c^ (ng g^−1^)	Concentration (ng g^−1^)	CV(%)^d^	RE(%)^e^	ME^f^ (%)	Recovery ± SD (%)
TC	0.5–500	0.9990	0.17	0.5	10	5.23	3.63	9.77	62.3 (6.8)
					100	4.25	2.19	9.67	91.8 (7.7)
					250	1.22	2.08	9.40	88.3 (12.5)
OTC	0.5–500	0.9996	0.17	0.5	10	4.98	−4.12	9.22	90.4 (2.8)
					100	3.59	−3.25	7.81	89.0 (3.3)
					250	2.41	−1.07	7.50	82.3 (12.0)
DOX	0.5–500	0.9963	0.17	0.5	10	2.15	−3.87	10.30	82.1 (4.5)
					100	1.24	−2.45	8.20	92.6 (3.9)
					250	1.10	− 1.23	7.11	90.4 (13.1)
SMX	0.5–500	0.9996	0.17	0.5	10	6.78	5.26	8.65	63.0 (9.9)
					100	4.52	4.11	5.67	61.1 (7.6)
					250	3.91	2.32	2.94	59.9 (7.1)
SFD	0.5–500	0.9997	0.17	0.5	10	7.45	6.54	5.22	69.8 (5.5)
					100	6.35	4.92	4.78	68.3 (7.6)
					250	2.17	2.14	3.51	64.4 (9.4)
CIP	0.5–500	0.9996	0.17	0.5	10	5.93	−4.97	4.80	77.3 (6.4)
					100	4.72	−3.25	4.47	76.4 (1.5)
					250	3.45	−2.14	3.11	69.6 (7.6)
LVF	0.5–500	0.9946	0.17	0.5	10	4.58	−3.65	10.0	77.0 (10.0)
					100	3.19	−2.78	9.07	69.7 (2.9)
					250	2.08	−1.12	7.40	77.2 (8.0)
ENF	0.5–500	0.9961	0.17	0.5	10	2.63	6.32	9.19	68.1 (10.3)
					100	1.47	5.24	8.04	85.5 (8.8)
					250	1.05	4.17	6.87	80.0 (8.3)
MET	0.5–500	0.9992	0.17	0.5	10	3.69	3.56	5.28	57.3 (8.0)
					100	2.41	2.41	5.04	64.9 (5.8)
					250	2.32	1.72	3.28	62.8 (8.0)
TRI	0.5–500	0.9960	0.17	0.5	10	6.27	−6.27	7.21	71.0 (6.7)
					100	5.30	−5.32	6.99	85.4 (6.8)
					250	1.47	−4.12	4.42	80.7 (7.0)
VAN	0.5–500	0.9997	0.17	0.5	10	4.78	−6.35	7.53	95.5 (2.4)
					100	3.25	−4.78	5.74	90.3 (6.4)
					250	2.96	−2.54	3.79	95.9 (12.3)
TYL	0.5–500	0.9996	0.17	0.5	10	5.63	3.25	9.01	91.3 (7.3)
					100	4.21	2.14	8.15	88.4 (5.8)
					250	3.14	1.36	4.48	95.3 (6.9)
CLR	0.5–500	0.9956	0.17	0.5	10	7.89	−5.41	6.04	75.7 (6.2)
					100	6.32	−4.32	4.88	88.6 (12.1)
					250	4.28	−1.67	4.02	80.1 (5.8)
CLD	0.5–500	0.9988	0.17	0.5	10	3.89	6.38	6.55	80.3 (3.5)
					100	3.24	4.72	5.88	84.8 (9.8)
					250	1.46	2.93	4.15	89.3 (7.9)

^a^R^2^—coefficient of determination.

^b^LOD—limit of detection.

^c^LOQ—limit of quantification.

^d^CV—coefficient of variation.

^e^RE—relative error.

^f^ME—matrix effect.

The limit of detection was 0.5 ng g^−1^, and the limit of quantification was 0.17 ng g^−1^ for all analytes.

### Determination of AMs in soil and pore water samples

**Figure 3:**
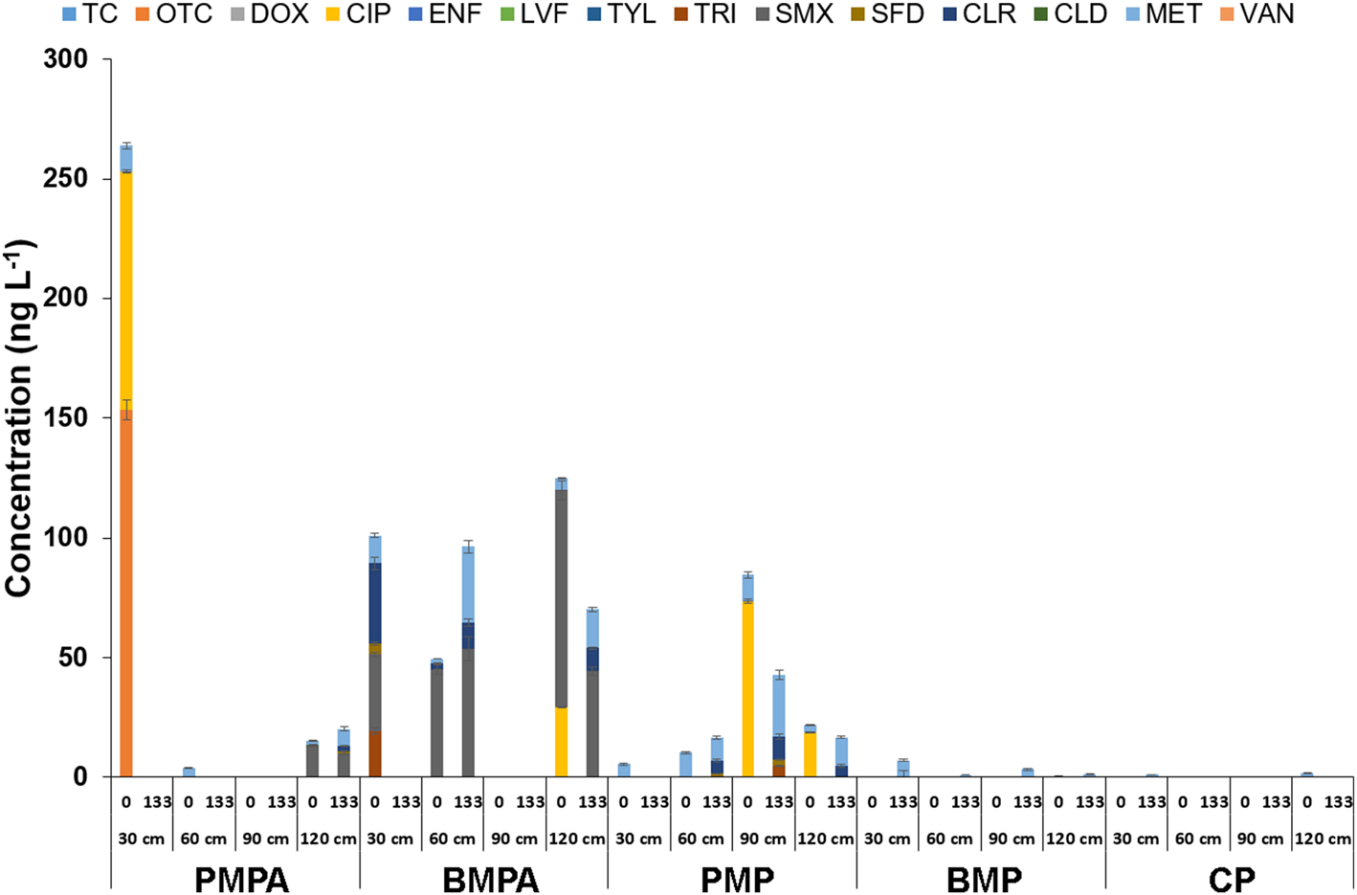
Antimicrobial agents content in pore water before soil enrichment AMs (A) on day 0 and (B) on the last day of the experiment (after 133 days) at different sampling depths (30–120 cm) (PMPA—soil plots supplemented with poultry manure and AMs; BMPA—soil plots supplemented with bovine manure and AMs; PMP—poultry manure soil plots; BMP—bovine manure soil plots; CP—control plots without supplementation (manure or AMs).

The results of the soil pore water samples are shown in Fig. 3 and those for the soil in Table 3.

**Table 3:** Concentrations of selected antimicrobials in soil samples fertilized with manure (ng g^−1^, dry weight).

Concentration (SD)															
**Samle**	Plot-Day	TC	OTC	DOX	CIP	ENF	LVF	MET	CLR	CLD	SMX	SFD	TYL	TRI	VAN
PMPA (soil plots supplemented with poultry manure and AMs)	1–0	nd	7.9 (0.8)	nd	8.1 (0.5)	nd	nd	nd	12.1 (1.8)	nd	nd	2.7 (0.4)	nd	nd	nd
	1–1	nd	5.6 (0.4)	73.4 (4.8)	8.3 (0.3)	55.8 (1.9)	nd	nd	5.6 (0.8)	nd	10.0 (1.0)	4.3 (0.6)	12.8 (1.1)	nd	nd
	1–14	nd	nd	66.8 (11.7)	8.1 (0.4)	70.3 (6.6)	nd	nd	nd	nd	8.1 (0.9)	3.0 (0.7)	8.2 (0.6)	nd	nd
	1–133	nd	nd	52.0 (8.1)	8.7 (0.2)	83.5 (11.4)	nd	nd	nd	nd	7.9 (0.8)	2.6 (0.4)	2.4 (0.3)	nd	nd
BMPA (soil plots supplemented with bovine manure and AMs)	2–0	nd	nd	nd	6.7 (0.2)	nd	nd	nd	nd	nd	nd	3.8 (0.5)	nd	nd	nd
	2–1	nd	nd	109.1 (14.4)	6.8 (0.3)	96.2 (3.4)	nd	nd	nd	nd	32.4 (5.1)	3.4 (0.5)	17.5 (1.4)	nd	nd
	2–14	nd	nd	48.2 (6.5)	7.0 (0.3)	107.1 (15.5)	nd	nd	nd	nd	25.7 (2.1)	4.3 (0.4)	13.8 (1.5)	nd	nd
	2–133	nd	nd	19.8 (13.8)	7.5 (0.2)	85.3 (10.4)	nd	nd	nd	nd	12.9 (1.3)	4.3 (0.8)	1.3 (0.1)	nd	nd
PMP (poultry manure soil plots)	3–0	nd	nd	nd	nd	nd	nd	nd	nd	nd	nd	nd	nd	nd	nd
	3–1	nd	nd	nd	1.7 (0.1)	nd	nd	nd	nd	nd	nd	3.7 (0.3)	nd	nd	nd
	3–14	nd	nd	nd	2.1 (0.3)	nd	nd	nd	nd	nd	nd	2.4 (0.2)	nd	nd	nd
	3–133	nd	nd	nd	0.7 (0.1)	nd	nd	nd	nd	nd	nd	4.0 (0.5)	nd	nd	nd
BMP (bovine manure soil plots)	4–0	nd	nd	nd	nd	nd	nd	nd	nd	nd	nd	nd	nd	nd	nd
	4–1	nd	nd	nd	nd	nd	nd	nd	nd	nd	nd	nd	nd	nd	nd
	4–14	nd	nd	nd	nd	nd	nd	nd	nd	nd	nd	nd	nd	nd	nd
	4–133	nd	nd	nd	nd	nd	nd	nd	nd	nd	nd	nd	nd	nd	nd
CP (control plots without supplementation)	5–0	nd	nd	nd	nd	nd	nd	nd	nd	nd	nd	nd	nd	nd	nd
	5–1	nd	nd	nd	nd	nd	nd	nd	nd	nd	nd	nd	nd	nd	nd
	5–14	nd	nd	nd	nd	nd	nd	nd	nd	nd	nd	nd	nd	nd	nd
	5–133	nd	nd	nd	nd	nd	nd	nd	nd	nd	nd	nd	nd	nd	nd

*nd—not detected.

Although the selected experimental plots were never fertilized with animal manure, trace amounts of 8 out of 14 AMs were detected in the pore water collected before the experiment (May). OTC, CIP, TRI, SMX, SFD, CLR, CLD, and MET were detected randomly. It means that initially, the soil from which pore water was taken was heterogeneous and was contaminated from additional sources. CIP was detected in poultry manure at a concentration of 30.03 ± 2.52 ng g^−1^, while in cattle manure, none of the 14 AMs was detected. Low concentration or lack of AMs was the reason why manure was enriched with a mixture of DOX, SMX, TYL, and ENF with a concentration of 50 µg g^−1^. The presence of selected AMs in pore water samples was most likely related to surface runoff from adjacent agricultural areas where animal manure was applied. Due to runoff from fields during heavy rainfall, AMs are mobile and can spread through the environment. Only two AMs introduced with soil and manure—SMX and CIP—have also been detected in pore water. The concentrations of detected AMs in pore water are shown in Fig. 3. The most common pollutant was MET, which was found in pore water collected from all plots at concentrations ranging from 0.4 to 11.5 ng L^−1^. MET was detected at all depths, which indicates it is mobile in soil. CIP was an equally common contaminant and was detected in pore water from PMPA, BMPA, and PMP in the range 18.7–99.6 ng L^−1^ (Fig. 3). The mobility of CIP in the environment and the factors influencing its leaching from soil to pore water have been the subject of many studies^40–42^. On the last day of the experiment, CIP was not detected in pore water samples, which may suggest its absorption into the soil. A high concentration of OTC (153.5 ng L^−1^) was detected only in the samples taken from PMPA, and in the samples from BMPA, only TRI was detected (19.3 ng L^−1^). According to the literature, OTC is a compound with a strong sorption affinity (*K*_OC_ 417–1026 mL g^−1^) and low mobility, which may result in a high concentration of the AMs in one place^43,44^. In pore water from PMPA, BMP, and CP, traces of CLD were detected at depths of 90 and 120 cm. CLR was detected only in pore water from BMPA, at depths of 30 and 60 cm. Among the SAs, SMX (13.2–90.8 ng L^−1^) was detected in the highest concentration in 4 out of 20 pore water samples. SFD was detected only at a depth of 120 cm in PMPA. SMX and MET are highly soluble and mobile in soil^43,45^. It was noticeable as they were detected at different depths at the exact sampling location. Trace amounts of AMs residues in pore water are reported worldwide. In a study conducted in Pakistan, the mean concentration of AMs was 2.3–13.8 ng L^−1^, with CIP being the most frequently detected compound^46^. Research conducted in Korea confirmed the widespread presence of FQs (0.2–0.7 ng L^−1^) and SAs (0.2–0.5 ng L^−1^) in pore water, but they were present only in rural agricultural areas; however, they were not detected in non-agricultural areas^47^.

On the last day of the experiment, 6 of the 14 target AMs were detected in the pore water (CIP, TRI, SMX, SFD, CLR, and MET). OTC and CLD residues were not detected at any of the water sampling depths. It has been shown that OTC and CLD can degrade under natural conditions under the influence of pH^48,49^. MET was detected in samples collected from all experimental plots, and its content ranged from 0.2 to 31.6 ng L^−1^, demonstrating its high stability in the environment. SFD was detected in 4 of 20 analyzed samples, and its concentration ranged from 0.27 to 2.49 ng L^−1^. CLR was detected in 6 of 20 analyzed samples in PMPA, BMPA, and PMP. This result differs from the sampling on day 0 as CLR was only detected in the pore water in BMPA. It can be seen that during the 133 days of the experiment, CLR dispersed in the soil and was detected in pore water taken at all depths except 30 cm. The CLR content of the pore water samples was in the range of 2.3–11.0 ng L^−1^. According to the literature, CLR is a pollutant that adsorbs strongly to soil and should not easily leach into pore water. However, it has been shown that sorption to soil (and therefore mobility) largely depends on its properties (pH, number of available sorption sites, and sorption complex saturation), which may explain the dispersion of CLR in soil^50,51^. TRI was not detected in the pore water samples from BMPA as on day 0, but it was detected in the samples from PMP (4.8 ng L^−1^).

One of the eight detected AM agents (SMX) was also introduced with enriched manure into the environment. SMX was also detected after 133 days of experiment in samples from PMPA at a depth of 120 cm and BMPA at a depth of 60 and 90 cm. It can be seen that compared with the pore water samples collected on day 0, on day 133 of the experiment, no SMX was detected at a depth of 30 cm, but a higher concentration of SMX (53.7 ng L^−1^) was detected in BMPA at a depth of 60 cm. SMX was introduced into the soil along with bovine manure, which may explain the increase in its content in pore water samples. In the case of samples taken at a depth of 120 cm, a decrease in the SMX concentration was observed.

Even though the soil was never fertilized with manure-based fertilizer, residues of AM agents were detected in it. Table 3 presents the evolution of concentration AMs over 133 days. Trace amounts of four AMs (OTC, CIP, SFD, and CLR) were detected in soil samples collected before the experiment. As in pore water, OTC was detected at concentrations of 5.6–7.9 ng g^−1^ only in PMPA. OTC was not detected in the samples after 14 days or after the end of the experiment. According to the literature, the half-life of OTC in manure is 30 days, but it can be detected in samples even five months after collection^22^. Additionally, when introduced into the environment, it strongly absorbs and accumulates in the soil^43^.

CIP trace amounts (1.7–8.7 ng g^−1^) were detected in soil samples from PMPA, BMPA, and PMP. The low pH of the soil promoted CIP absorption, and this mechanism is based on cation exchange and coordination with organic matter^40^. Moreover, the content and type of humic acids (HA) are also important, as they affect the sorption capacity of the soil and the concentration of divalent cations^41^. The concentration of CIP in the soil remained constant throughout the experiment, which proves its stability under environmental conditions. CIP is a common contaminant in environmental samples because it is a primary AM and also the main metabolite of ENF, which is widely used in veterinary medicine^52^.

Similarly, SFD was detected in the same soil samples (PMPA, BMPA, PMP) in the range of 2.4–4.3 ng g^−1^ and 0.7–4.6 ng L^−1^ in pore water. CLR was only detected in samples from PMPA, and its concentration dropped by 50% after just one day. CLR has not been detected in other types of soil samples. This contamination can come from many sources, such as livestock feces and runoff from surrounding farmland.

None of the four AMs selected for the experiment (DOX, ENF SMX, or TYL) were detected in the soil samples collected before enrichment (day 0). Comparing PMPA and BMPA, it was noticed that significantly higher concentrations of analytes were detected in the bovine manure samples. It was caused due to the method of soil fertilization, where 4 kg of manure was used per 1.0 m^2^ of soil, and in the case of poultry manure, it was 1.5 kg m^−2^.

The highest concentration of DOX was detected on the day of supplementation with manure, which was 109.1 ng g^−1^ for poultry and 73.4 ng g^−1^ for bovine. After 14 days of the experiment, the DOX content in the soils decreased. The soil fertilized with poultry manure contained 66.8 ng g^−1^ DOX (8% decrease), while in the soil fertilized with bovine manure, 48.2 ng g^−1^ was detected (55.8% decrease). The degradation rate of the AMs was significantly faster in soils enriched with bovine manure. In the samples collected on the 133rd day of the experiment, the concentration of DOX in the soil collected from PMPA was 52.0 ng g^−1^, and in BMPA, it was 19.8 ng g^−1^. This means that in samples with bovine manure, the loss of DOX was 81.8%. It can be seen that in soil samples fertilized with poultry manure, taking into account the standard deviations, the DOX concentration remained constant throughout the experiment. DOX was not detected in pore water samples, suggesting that it is not mobile, so the decrease in its content may be related to biotic environmental degradation. The same trend was observed for SMX, in which the concentration of SMX in poultry manure was constant (7.9–10.0 ng g^−1^), but in bovine manure, it decreased from 32.4 ng g^−1^ to 12.9 ng g^−1^ within 133 days. SMX was also detected in pore water on days 1 and 133 of sampling, which proves its mobility in the environment. A systematic decrease in the TYL content was observed in both types of soil samples during the experiment. ENF was present in soil samples in the range 55.8–107.1 ng g^−1^. Taking into account the standard deviations from the average ENF content in the soil, it can be seen that its concentration did not change significantly during the experiment. In the samples from PMPA, there was a slight increase in the ENF content from 55.8 to 83.5 ng g^−1^. It could be caused by atmospheric factors such as soil temperature and moisture, which could have increased the sorption and accumulation of the AMs in the soil^7,42^.

The addition of animal manure may affect the physicochemical properties of the soil due to the higher content of organic matter and water compared to soil (Table S3). These parameters influence pharmaceuticals' sorption properties and their mobility in soil^53^. The effect of addition animal excrement on the sorption constant was investigated using the group of SAs. The SAs sorption depends on the fertilizer ratio applied to the soil^54^. For example, the mobility of SMX and SFD in the soil varies and is correlated with the organic carbon content in the soil, the inorganic compound content, and hydrophobic interactions^7^. In our research, the presence of SAs was noted in both pore water and soil samples, which means that they gradually leached from the soil. A different mechanism is observed by substances, i.e., DOX, CIP, and ENF. The concentrations of CIP and ENF did not change in the tested matrices during the experiment. However, FQs sorption is not correlated with the organic carbon content in the soil, but the dominant mechanism is cation exchange^7^. ENF, CIP, and DOX are highly persistent in soil and do not leach easily^9^.

Summing up, it was noticed that after the introduction of DOX, TYL, and ENF into the soil water system, they were adsorbed by the soil. According to the literature, ENF, TYL, and DOX accumulate in the soil and are not easily washed away^9,43^. These 3 AMs were not detected in the pore water, so it can be assumed that the decrease in their content during the experiment was unrelated to washing out by rain deeper into the soil. SMX was present in the pore water in PMPA and BMPA before the start of the experiment in the concentration range of 13.2–90.8 ng g^−1^, which indicates its high prevalence in the environment. At the end of the experiment, a higher SMX concentration was observed in pore water collected from BMPA at a depth of 60 cm. On the other hand, for PMPA and BMPA, a decrease in the SMX content was observed in the pore water collected at a depth of 120 cm. Trace amounts of OTC, CIP, MET, TRI, SFD, CLR, and CLD dispersed in the studied water-soil system were identified. However, the source and soil dispersion of these AMs is unknown therefore, no broad conclusions can be drawn about their mobility in the soil–water system. The stability of AMs for four months in soil and water is a disturbing phenomenon, potentially dangerous to human health. Residues of AMs in soil may promote the development and spread of antibiotic resistance genes^8,55^. Moreover, plants can absorb AMs from the pore water and re-introduce them into the human digestive system^56,57^. AMs residues affect soil respiration because they reduce the diversity and abundance of bacteria, which indirectly changes the stoichiometry of soil nutrients^58,59^.

### Identification AMs TPs in environmental samples

The structures of the identified TPs are presented in Table 4. In soil samples enriched with the TYL standard, four TPs were detected, marked by the literature with letters: B (desmycosin), C (macrocin), D (relomycin), and tylosin A aldol (TAD). TYL transformation products were not detected in soil samples collected before fertilization with AMs enriched manure or in control plots (PMP and CP). The presence of TYL B (m/z 772) was confirmed based on the fragmentation ion signals at m/z 174 and 156. For TYL D (m/z 918), fragmentation signals of m/z 174 and 425 were observed. TYL A Aldol with the same m/z as TYL A was confirmed based on the two signals at m/z 772 and 598, which were not present in the fragmentation path of TYL A. TYL B and D and TAD were detected in both samples fertilized with poultry manure supplemented with AMs (PMPA) and bovine manure supplemented with AMs (BMPA). TYL C (m/z 902) was identified only in samples of bovine manure enriched with AMs. Two fragmentation ions confirmed its presence at m/z 174 and 460. The described TPs fragmentation pathways have been established in the literature: TAD^60^, TYL B^61^, TYL C, and TYL D^62,63^. All TPs are products of the natural degradation of TYL in the environment, and their occurrence has been described in various matrices, including honey^61^, surface water^64,65^, and pig manure^66^. TYL TPs have not been detected in pore water.

**Table 4:** Proposed structures of TPs of selected antimicrobials.

Abbrev.	[M+H]^+^	Proposed structure	Identified in samples
CIP	332.3		PMPA BMPA PMP
ENF	360.4		PMPA BMPA
ENF_1	263.2		PMPA BMPA PMP
ENF_2	292.0		PMPA PMP
ENF_3	360.0		PMPA BMPA
ENF_4	291.0		PMPA BMPA PMP
ENF_5	334.0		PMPA BMPA PMP
MET	172.7		PMPA BMPA PMP BMP CP
MET_1	143.0		BMPA
SMX	253.9		PMPA BMPA
SMX_1	156.0		BMPA
SMX_2	288.2		PMPA
SMX_3	244.0		PMPA BMPA
TYL	916.2		PMPA BMPA
TYL B	772.4		PMPA BMPA
TYL C	902.4		BMPA
TYL D	918.4		PMPA BMPA
TAD	916.0		PMPA BMPA
DOX	445.0		PMPA BMPA
DOX_1	432.0		PMPA BMPA
DOX_2	477.0		PMPA BMPA

PMPA—soil plots supplemented with poultry manure and antimicrobials; BMPA—soil plots supplemented with bovine manure and antimicrobials; PMP—poultry manure soil plots; BMP—bovine manure soil plots; CP—control plots without supplementation (manure or antimicrobials).

Among the FQs, ENF was introduced into soil samples and manure, but CIP was detected in both types of samples (liquid and solid) taken before the experiment. As is known, CIP is an ENF metabolite that differs structurally, with an ethyl group attached to the nitrogen atom in the piperazine ring^67^; therefore, it is not possible to determine whether the detected TPs arose directly from the ENF or further CIP degradation. Five TPs of ENF were detected in soil and pore water extracts samples. The first, ENF_1, resulted from the detachment of the piperazine ring from the ENF molecule, with the formation of TPs with m/z 263. Fragmentation ions with m/z 245 and 204 were recorded in the ENF_1 mass spectrum, which aligns with the transformation path described in the literature^68^. It is the most frequently detected TP of ENF and CIP and has been recorded during sewage treatment^69,70^ and microbiological degradation^71^. Another product, ENF_2 with m/z 292, can be formed in two methods: indirectly from ENF or directly from its metabolite, CIP. In the second method, ENF_2 is generated by the detachment of the cyclopropyl ring from CIP. A characteristic fragmentation ion at m/z 274 was observed in the mass spectrum. This TP formed during the degradation of ENF in aqueous samples due to UV radiation^69^. ENF_3 (m/z 360) was identified by two characteristic fragmentation ions, m/z 342 and 318, as described in the literature. This product was identified in soil samples fertilized with doped manure. ENF_3 was formed by the attachment of a carbonyl group to the nitrogen atom in the piperazine ring. ENF_3 was described as a product of the CIP transformation, arising from biotic transformations^72^ and UV radiation^73^. ENF_4 (m/z 291) was formed by carbonylation of the amino group on the quinolone ring of ENF_1. Ions at m/z 273 and 217 were observed in the ENF_4 mass spectrum, which confirmed the structure of compound^74^. ENF_5 (m/z 334) was formed by opening the piperazine ring with the loss of C_2_H_2_ and the attachment of the carbonyl group to nitrogen. These TPs have been shown to create during the photolytic^68,74^ and the microorganisms degradation^72^ of CIP or ENF. ENF_5 was identified based on two transitions from m/z 334 to m/z 316 and 245 described in the literature.

Three SMX TPs were detected, resulting from S–N bond cleavage, hydroxylation, and ring-opening reactions. SMX_1 is the simplest TP formed from the degradation of SMX, resulting from the breakage of the S–N bond to form a 4-aminophenylsulfonyl structure. This compound has an m/z of 156 and fragment ions of m/z 108 and 65 in the mass spectrum^75^. SMX_2 (m/z 288) resulted from the double hydroxylation of the isoxazole ring. Fragmentation ions at m/z = 270, 188, and 160 were observed in the mass spectrum, which is in line with the fragmentation path of this compound described in the literature^76^. The last product, SMX_3 (m/z 244), was formed as a result of isoxazole ring-opening and its oxidation. The compound transformation path has been described in model conditions during the electrochemical oxidation of SMX^77^, but it cannot be ruled out that it may be formed due to environmental factors. The compound was identified by recording the mass spectrum, in which ions at m/z 156 and 108 were observed.

The DOX degradation pathways under environmental conditions have not been fully described. In general, DOX degradation is carried out under model conditions^53,78^. The first TP of DOX detected in soil extracts was a compound with m/z 432 (DOX_1). The mass spectrum showed fragment ions with m/z 415 [M + H–NH_3_]^+^, 371 [M + H–NHCO–H_2_O]^+^, and 327 [M + H–NHCO–H_2_O–N(CH_3_)_2_]^+^. This compound is formed due to DOX dehydration. This compound has been detected in catalytic oxidation in water^79^, but the loss of H_2_O can also occur under environmental conditions. DOX_2 was formed as a result of the double hydroxylation of the ring at positions 2 and 6. An intense fragmentation ion of m/z 460 (−NH_2_ loss) and an ion of m/z 416 (-CO loss) were observed in the mass spectrum, which is consistent with the fragmentation path of this compound in the literature^80^.

MET was detected in all experimental plots in pore water, and one MET TP was detected. MET_1 was formed due to MET hydroxylation in the presence of humic acid^81^. This product was also created as a result of MET degradation in aquatic environments under the influence of UVA radiation^82^. The structure of MET_1 (m/z 143) was confirmed by the presence of two fragmentation ions with m/z 125 and 97 in the mass spectrum. In the literature, MET_1 is formed mainly due to reactions with UV radiation, but based on our research, it can be concluded that this product can also be created directly in the environment. Under environmental conditions, the soil surface is constantly exposed to sunlight, which will lead to the formation of MET_1.

To summarize, the TP of MET was detected in all experimental plots. The TPs DOX, TYL, and SMX were detected only in samples (water and soil) from plots enriched with manure with selected AMs. Two TPs of FQs were detected in PMP, in soil fertilized with poultry manure: ENF_1 and ENF_2. The presence of these two TPs was not directly related to ENF transformation. ENF_1 and ENF_2 were produced from the degradation of CIP residues detected in PMP. The remaining transformation products of FQs were detected only in PMPA and BMPA. Non-targeted analysis was also performed for CLR, SFD, and OTC, but no TPs were detected for them.

## Conclusion

As part of this research, an SLE-SPE-LC–MS/MS procedure for determining selected AMs in soil samples was developed, validated, and applied to environmental samples. Due to the soil's co-elution of humic and fulvic acids, two SPE cartridges were combined in tandem. The developed procedure allowed for the extraction of analytes from the soil with an efficiency of 57–96% with good repeatability. Additionally, SPE-LC–MS/MS procedure was used to analyze AMs in soil pore water samples.

Trace amounts of OTC, CIP, TRI, SMX, SFD, CLR, CLD, and MET were detected at different sampling depths in pore water samples collected on the zero day of the experiment (before fertilization with manure containing AMs). Six of these AMs were also present in pore water on the experiment's last day, proving their persistence. Of the four AMs introduced into the soil, only SMX was present in pore water samples at depths from 60 to 120 cm on day 133 of the experiment, which proves its mobility after it enters the soil.

All supplemented AMs (DOX, SMX, TYL, and ENF) were determined in soil extract samples. Over time, DOX, SMX, and TYL concentrations in the samples decreased, while ENF remained constant. Moreover, additional pharmaceutical contaminants (CIP, SFD, and CLR) were detected in soil samples.

A non-targeted analysis was also performed, in which 16 TPs were detected for DOX (2), SMX (3), TYL (4), ENF (6), and MET (1). All products of the transformation of AMs were detected in the plots enriched with the mixture of AMs. Additionally, the TPs of ENF were detected in the control plots. Generally, described TPs were formed as a result of carbonylation, oxidation, and ring-opening reactions that may have occurred due to biotic and abiotic transformations under environmental conditions.

## Supplementary Information

Supplementary Information.

## Data Availability

All data generated and analyzed during our study are included in this article.
