# Supplementary material for: Determination of antimicrobial agents and their transformation products in an agricultural water-soil system modified with manure
Source: Sci Rep. 2022 Oct 20;12:17529. doi: 10.1038/s41598-022-22440-5 (PMC9584908; doi:10.1038/s41598-022-22440-5)
Supplement: Supplementary file 1 — Supplementary Information. [file 41598_2022_22440_MOESM1_ESM.docx]

**Determination of antimicrobial agents and their transformation products in an agricultural water-soil system
modified with manure**

Klaudia Stando^1^, Ewa Korzeniewska^2^, Ewa Felis^3,4^, Monika Harnisz^2^,
Martyna Buta-Hubeny^2^, Sylwia Bajkacz^1,3^*

^1^ Silesian University of Technology, Faculty of Chemistry, **Department of Inorganic, Analytical Chemistry and Electrochemistry**, B. Krzywoustego 6 Str., 44-100 Gliwice, Poland

^2^ University of Warmia and Mazury in Olsztyn, Faculty of Geoengineering, Department of Engineering of Water Protection and Environmental Microbiology, Prawocheńskiego 1 Str., 10-720 Olsztyn, Poland

^3^ Silesian University of Technology, Centre for Biotechnology, ul. B. Krzywoustego 8, 44-100 Gliwice, Poland

^4^ Silesian University of Technology, Faculty of Power and Environmental Engineering, Environmental Biotechnology Department, Akademicka 2 Str., 44-100 Gliwice, Poland

Corresponding author: Sylwia Bajkacz (e-mail: sylwia.bajkacz@polsl.pl)

**SUPPLEMENTARY MATERIAL**

**Summary**

This supporting information file includes additional results and information as described in the text of the main article including:

**S1. Supplementation to section** 2.1 Standards, chemicals and materials

**S2. Supplementation to section**:2.3 Sample preparation

**S3. Supplementation to section** 2.5 Method validation

**S4. Supplementation to section 3.1** Step by step to the effective extraction procedure – development of the SLE-SPE conditions for extraction of AMs from soil samples

**S5. Supplementation to section S5**. Method validation

**Table S1.** Extraction methods and determination of pharmaceutical residues in soil, manure, and sediment – review

**Table S2.** MRM (multiple reaction monitoring) parameters of the antimicrobials analyzed in this study **Table S3.** Selected physical and chemical parameters of various matrices used in the experiment

**Table S4.** Recovery of analytes using various types of SPE (solid-phase extraction) cartridges

**Figure S1.** Stability of the analytes in the solvent during different times of shaking (30 min and 60 min)

**Figure S2.** Effect of UAE on the extraction efficiency (5–30 min; extraction mixture: citrate buffer (pH=4):acetonitrile (1:1; v/v))

**Figure S3.** Influence of matrix (comparison of pure solvent and soil extract) on the efficiency of the SPE procedure (solvent 1: citrate buffer (pH=4):acetonitrile (1:1; v/v); solvent 2: 0.2 M sodium hydroxide:acetone (1:1; v/v)

**Figure S4.** Effect of type of matrix (soil samples and quartz sand) on the efficiency of the SPE procedure

**Figure S5.** Effect of evaporation conditions on the recovery of AMs

**Figure S6.** Effect of the solvents used for the dissolved of residues after SLE-SPE procedure on the recovery of analytes

**Figure S7.** Effect of the filter materials on the recovery of analytes

## S1. Supplementation to section 2.1 Standards, chemicals and materials

Analytical standards of tetracycline (TC), doxycycline (DOX), oxytetracycline (OTC), ciprofloxacin (CIP), enrofloxacin (ENF), levofloxacin (LVF), metronidazole (MET), tylosin (TYL), trimethoprim (TRI), vancomycin (VAN), clarithromycin (CLR), clindamycin (CLD), sulfamethoxazole (SMX) and sulfadiazine (SFD) were purchased from Sigma-Aldrich (St. Louis, MO, USA). Analytical-grade acetonitrile, methanol, acetone, formic acid, hydrochloric acid, citric acid, a 25% ammonium hydroxide solution, disodium phosphate, sodium hydroxide, ethyl acetate, ethylenediaminetetraacetic acid disodium salt (EDTA) were purchased from CHEMPUR (Piekary Śląskie, Poland). Analytical-grade citrate buffer (pH=3.0, pH=4.0) was purchased from Merck (Darmstadt, Germany). Hypergrade water, acetonitrile, and formic acid were obtained from Merck (Darmstadt, Germany) and used for the LC-MS analyses. Oasis HLB cartridges (500 mg, 6 mL) and Oasis WAX (60 mg, 3 mL) from Waters (Eschborn, Germany), Bond Elut Plexa (60 mg, 3 mL) from Agilent (Santa Clara, CA, US), and Bakerbond SPE^TM^ C18 (60 mg, 3 mL) from Avantor Performance Materials POLAND S.A. (Gliwice, Poland) were used for solid-phase extraction. Quartz sand with 0.4-1.5 mm was purchased from Kreisel (Dąbrowa Górnicza, Poland).

McIlvaine buffer solution (pH=4) was prepared by mixing 61.5 mL of 0.1 M citric acid with 38.5 mL of 0.2 M disodium hydrogen phosphate. pH was adjusted to pH 4.0±0.1 with citric acid or disodium hydrogen phosphate, as stated in recipe ^1^. A fresh buffer solution was prepared before each extraction.

The standard stock solutions of TC, OTC, DOX, TYL, CLD, ENF, MET, SMX, TRI, CLR, and LVF with a concentration of 1.0 mg mL^-1^ were diluted in methanol. CIP was dissolved in 0.1% formic acid in methanol, SFD in acetone, and VAN in methanol:water (1:1; v/v). Working solutions of the AMs were prepared in the range of 1.0 – 600.0 ng mL^-1^. The working solutions with defined concentrations were added to the blank samples (soil without selected AMs) 24 h before analysis. The quality control (QC) samples were prepared at three concentration levels: low-quality control (LQC=10 ng g^-1^), middle-quality control (MQC=100.0 ng g^-1^), and high-quality control (HQC=250 ng g^-1^). Stock solutions were stored for one month, while working solutions were stored for a maximum of one week. All solutions were stored in the dark at 4 °C.

## S2. Supplementation to section:2.3 Sample preparation

## S2.1 SLE procedure

***a) Selection solvent and time***

A single SLE extraction efficiency was tested, enriching 2.0 g of soil with 5 µg of antimicrobials standards solution. Then 10 mL of extraction solvent was added and shaken for 30 min at 900 rpm. The samples were centrifuged, and 1.0 mL of supernatant was collected (the maximum concentration of analytes in the supernatant was 500 ng mL^-1^). The result was related to an AMs standard with a concentration of 500 ng mL^-1^ prepared in the same solvent.

In the next step, the stability of AMs in the selected solvent was checked. The shaking time was checked after selecting three of the best extraction solvents, such as: citrate buffer (pH=4):acetonitrile (1:1; v/v), McIlvaine buffer (pH=4):methanol (1:1; v/v), 0.2 M sodium hydroxide:acetone (1:1; v/v). The experiment was performed by adding 500 ng of the antimicrobials standard solution to 1 mL of the selected solvent and shaking for 30 or 60 min at 900 rpm at room temperature.

***b) Application of ultrasound-assigned extraction***

A mixture of selected AMs was prepared at a concentration of 500 ng mL^-1^ in methanol and placed in an ultrasonic bath to check the stability of analytes under ultrasound-assigned extraction (UAE). Antimicrobials solutions were treated with ultrasound for: 0, 5, 10, 15, 20, and 30 min. Samples were prepared in triplicate and analyzed in triplicate. The results were obtained by comparing the surface area of the analytes after the treatment with ultrasound with the non-sonicated sample (0 min).

***S2.2 SPE procedure***

***a) Selection of solvent used to dilution extract before SPE***

The solvent in which the samples were dissolved/diluted and loaded to an OASIS HLB cartridge (500 mg, 6 mL) was selected. The experiment had two variants - (I) with the evaporation of the solvent after SLE and redissolution of the residue or (II) with a dilution of the solvent immediately after SLE. For the first variant, 20 mL solvent (0.1% FA, H_2_O or MeOH:H_2_O (5:95; v/v)) were checked. For the second variant, 20 mL solvent (McIlvaine buffer (pH=4):methanol (1:1; v/v); McIlvaine buffer (pH=4): acetonitrile (1:1; v/v) and 0.2 M sodium hydroxide solution:acetone (1:1; v/v)) were prepared and diluted with 200 mL of distilled water, enriched with a standards mixture and adjusted to pH=3. The obtained samples were passed through the HLB cartridge, then the cartridges were vacuum dried for 20 minutes, and elution was performed with 6 mL of methanol.

***b) The influence of the soil matrix on the efficiency of the SPE procedure***

To study the effect of matrix components on the efficiency of SPE extraction,a "matrix solution" was prepared using two solvents (Solvent 1: citrate buffer (pH=4):acetonitrile (1:1; v/v) and Solvent 2: 0.2 M sodium hydroxide:acetone (1:1; v/v)). By "matrix solution" it means the supernatant after SLE extraction of the soil without AMs. A matrix solution was prepared as follows: To 2.0 g of soil not enriched with antimicrobials, 10 mL of the selected solvent was added, sonicated for 10 min, and shaken for 60 min at 900 rpm, followed by centrifugation for 10 min at 8000 rpm. The supernatant was transferred to a 250 mL glass bottle, and immediately before the SPE, it was enriched with 500 ng of the mixture of antimicrobials standards solution, diluted with 200 mL of distilled water, and adjusted to pH=4 using formic acid. OASIS HLB (500 mg, 6 mL) cartridges were used for SPE extraction. Cartridges after extraction were vacuum dried for 20 min, then analytes were eluted with 12 mL of methanol and evaporated to dryness. Samples without matrix components were prepared by analogy. 20 mL of the selected solvent was enriched with 500 ng of standard and diluted with 200 mL of water. The next steps were the same as described above.

The effect of soil organic matter on the efficiency of analytes extraction was verified after the application of single SLE extraction with three selected solvents: citrate buffer (pH=4):acetonitrile (1:1; v/v), 0.2 M sodium hydroxide:acetone (1:1; v/v), McIlvaine buffer (pH=4):methanol (1:1; v/v), followed SPE purification. The quartz sand and soil extracts were prepared as follows: on 2.0 g of purified quartz sand, 500 ng of antimicrobials standard solution were applied and left for 24 hours. 10 mL of solvent was added to the sample, sonicated
10 min, shaken 60 min at 900 rpm. The samples were centrifuged, and the supernatant was diluted with 200 mL of distilled water and adjusted to pH=4. OASIS HLB cartridges (500 mg, 6 mL), conditioned with methanol,
0.1 M hydrochloric acid, and distilled water, after sample loading, the sorbent was dried, and then analytes were eluted with 12 mL of methanol.

***c) Selection of tandem-SPE conditions***

Development of a tandem SPE purification procedure began with the selection of a pre-column. Three types of SPE cartridges were tested: Bond Elut Plexa, Waters C18, and OASIS WAX with the same parameters (60 mg, 3 mL). A soil matrix extract prepared in 0.2 M sodium hydroxide:acetone (1:1; v/v), enriched with analytes, diluted to 200 mL of water, and adjusted to pH=4, was applied to each of the columns. Elution was performed with 6 mL of methanol.

In the next step, OASIS WAX (60 mg, 3 mL) was combined with OASIS HLB (500 mg, 6 mL), and the matrix solution was prepared in two solvents (citrate buffer:acetonitrile (1:1; v/v) and McIlvaine buffer:methanol
(1: 1; v/v)) was serially passed through them. Before SPE matrix solution was analytes enriched, diluted with
200 mL of water and adjusted to pH=4.

To select the elution solvent, a soil matrix solution in McIlvaine buffer (pH=4):methanol (1:1; v/v), diluted with 200 mL of water, enriched with 500 ng of the antimicrobials standards, and adjusted to the appropriate pH with formic acid was used for the tests. The analytes were eluted with methanol, 0.1% acetic acid in methanol and 0.1% ammonia in methanol of various volumes.

***S2.3 evaporation, dissolving, and filtration***

***a) Evaporation of the extract***

500 ng of the antimicrobials mixture was added to a vial containing 15 mL of methanol and evaporated in the air or in nitrogen to study the influence of the evaporation conditions on the recovery of analytes.

***b) Dissolving residues***

500 ng of the antimicrobials mixture was added to a vial containing 15 mL of methanol and evaporated with a stream of nitrogen to select a solvent for dissolving residues before LC-MS/MS analysis. The residue was dissolved in the selected solvent by handshaking three times for one minute. Solvents were selected based on the literature as a mixture of acetonitrile, methanol, and acidified water.

***c)*** ***Filtration of the extract***

The mixture of standards (500 ng) dissolved in 1 mL of a mixture of 0.1% formic acid in water:methanol (1:1; v/v), was passed through nylon and polyethersulfone syringe filters, differing in size and porosity.

## S3. Supplementation to section 2.5 Method validation

The developed LC-MS/MS method for analysis of 14 antimicrobials in soil was validated. The analytical method was evaluated in terms of linearity, selectivity, sensitivity (LOD; limit of detection, LOQ; limit of quantification), matrix effect (ME), recovery, accuracy (RE; relative error), and precision (CV; coefficient of variation).

Linearity was determined based on the prepared calibration curves for the selected antimicrobials. The matrix solution was prepared by extracting soil samples not enriched with antimicrobials as described in Section 2.4.1. The calibration curves were in the range of 0.5 – 500 ng g^−1^. Regression equations for each analyte were obtained using the linear regression method, and then the coefficient of determination (R^2^) was determined. LOD and LOQ were calculated as the minimum detectable amount of analyte with a signal-to-noise ratio of 3 and 10, respectively. The matrix effects (ME) were evaluated by comparing the peak area of the analytes in matrix solution to the peak area of compounds diluted with a mixture of 0.1% formic acid in water:methanol (1:1; v/v).

The effectiveness of the developed extraction procedure was assessed based on the recovery value (RE). Soil samples were enriched with analytes at three concentration levels: LQC 20 ng g^-1^, MQC 100 ng g^-1^, and HQC 250 ng g^-1^. The enriched soil samples were then extracted according to the procedure given in section 2.4.1. Each of the samples was prepared in triplicate and analyzed in triplicate. Recovery was defined as the ratio of the measured signal analyte area in the sample after extraction related to the signal area of the matrix solution enriched with antimicrobials. Accuracy and precision were also determined for soil samples enriched at three concentration levels (LQC, MQC, HQC). Accuracy was defined as the relative error (RE) for analyzes performed in six replications. The precision of the method was determined to form on the coefficient of variation (CV).

The multi-reaction monitoring mode in the targeted analysis was used to obtain high selectivity. Blank soil samples were analyzed to determine the selectivity and specificity of the method. The absence of any chromatographic peak in soil matrix extracts at the same retention times as target compounds indicated that no matrix compounds were given a false positive signal.

**Table S1.** Extraction methods and determination of pharmaceutical residues in soil, manure, and sediment – review

| Analytes | Matrix | Matrix composition | Extraction | | Reconstituted | Analysis | Recovery  (%) | Ref. |
| --- | --- | --- | --- | --- | --- | --- | --- | --- |
|  |  |  | **SLE** | **SPE** |  |  |  |  |
| OTC | soil, manure | n.d | McIlvaine buffer  (pH=4), MeOH  10 min centrifugation  10351 rpm | MCAC columns  **C:** H_2_O, CuSO_4_ in H_2_O  **E:** MeOH, EDTA | n.d | HPLC-UV | **OTC** 79-81 | ^2^ |
| TYL |  |  | phosphate buffer  (pH=2.5):MeOH  (1:2; v/v)  10 min, centrifugation  10351 rpm | Waters C18 SPE  **C:** MeOH, H_2_O  **P:** H_2_O, 25% ACN  **E:** 1.25% AcA in MeOH | n.d |  | **TYL** 47-85 |  |
| SMX, SFD, CIP, ENF, DOX, TYL, TC, OTC, TRI | manure | n.d | Three-step extraction:  1. Na_2_EDTA- McIlvaine buffer (pH=4),  2. MeOH:ACN:Acetone (2:2:1; v/v/v)  3. n-hexane  ultrasonicated 10 min,  centrifugation 15 min,  4500 rpm | Tandem column:  Waters SAX  OASIS HLB  **C:** MeOH, H_2_O  **P:** H_2_O  **E:** MeOH:Acetone  (80:20 v/v)  **Ev:** N_2_, 50^o^C | 30% MeOH in H_2_O | UHPLC-MS/MS | **TCs** 59-73  **SAs** 63-80  **FQs** 54-68 | ^3^ |
|  | soil | n.d | MeOH:EDTA-citrate buffer  (3:2:19; v/v/v)  ultrasonicated 15 min,  centrifugation 5 min,  4000 rpm  Diluted: 200 mL H_2_O, pH=4 |  |  |  | **TCs** 60-81  **SAs** 65-90  **FQs** 61-73 |  |
| TYL, CLR  SMX | soil | **TOC**  22.6-68.0  mg g^-1^  **Sand**  9.5-26.1%  **Slit**  65.2-77.3%  **Clay**  8.7-14.3% | ACN:H_2_O (pH=7)  (20:15 v/v)  ultrasonicated 30 min  **Ev:** rotary evaporator, 40^o^C  diluted: 100 mL H_2_O | OASIS HLB  **C:** MeOH, H_2_O  **E:** MeOH  **Ev**: N_2_, RT | MeOH: 5 mM ammonium acetate  (3:2 v/v) | LC-MS/MS | **TYL** 135  (SD:20)  **SMX** 89  (SD:15)  **CLR** 115  (SD:5) | ^4^ |
| OTC, TYL | soil | **Sand**  42.6-85.3%  **Slit**  6.2-32.3%  **Clay**  8.5-25.1%  **OC**  1.3-3.5%  **CEC**  11.4-22.4% | MeOH:0.1 M EDTA:McIlvaine buffer (pH4)  (50:25:25 v/v/v)  ultrasonicated 10 min,  centrifugation 15 min,  3273 rpm  diluted: 400 mL H_2_O ,pH: 2.9 | Tandem column:  Waters SAX  OASIS HLB  **C:** MeOH, McIlvaine buffer (pH=4)  **P:** 0.1 M NaOAc, H_2_O, 20% MeOH  **E:** MeOH | - | LC-UV | **OTC** 38-75  **TYL** 47-105 | ^5^ |
| CIP, ENF, TC, OTC, SMX | soil | **Clay:**  32-36 %  **Slit:**  36-50 %  **Sand:**  14-32 %  **pH**  8.0  **OC:**  2.17-4.51 %  **CEC:**  18.06-19.15 cmol kg^-1^ | Potassium phosphate buffer (pH=3.2):ACN (1:1; v/v)  shaken: 20 min, 300 rpm  ultrasonicated: 10 min, 59 kHz  centrifugation: 10 min,  6000 rpm  repart: 2  diluted: 400 mL H_2_O | Tandem column:  Waters SAX  OASIS HLB  **C:** MeOH, H_2_O, potassium phosphate buffer (pH=3.2)  **P:** H_2_O  **E:** MeOH  **Ev:** N_2_, 30^o^C | MeOH | HPLC-MS/MS | **CIP** 67-74  **ENF** 65-77  **TC** 73-81  **OTC** 66-85  **SMX** 81-84 | ^6^ |
| DOX, ENF, SFD, TRI, TYL | soil  manure | n.d | MeOH:ACN:0.1 M EDTA: McIlvaine buffer (pH=4)  (30:20:25:25; v/v/v/v)  ultrasonicated: 10 min  centrifugation: 10 min,  4000 rpm  repart: 3  diluted: 500 mL H_2_O, pH=2.3 | OASIS HLB  **C:** MeOH, H_2_O  **P:** H_2_O  **E:** MeOH  **Ev:** N_2_, 45^o^C | 25% MeOH in 0.1% FA in H_2_O | LC-MS/MS | **DOX** 76-98  **ENF** 102-113  **SFD** 63-72  **TRI** 90-102  **TYL** 89-103 | ^7^ |
| ENF, CIP, OTC, DOX, TC, TYL, CLD | soil | n.d | Two-step extraction:  1. ACN:Na_2_EDTA-McIlvaine buffer (pH = 4.0)  (5:5; v/v)  2. 0.2 M NaOH  (adjusted to pH=4)  ultrasonicated: 10 min  shaken: 20 min  centrifugation: 10 min,  9000 rpm  **Ev:** water bath, 45^o^C | OASIS HLB  **C:** MeOH, H_2_O, Na_2_EDTA-McIlvaine buffer  **P:** 5% MeOH in H_2_O  **E:** MeOH  **Ev:** 45^o^C | 20% MeOH in 0.1% FA in H_2_O | LC-MS/MS | **ENF** 65-71  **CIP** 62-79  **TC** 54-67  **OTC** 68-71  **DOX** 65-71  **TYL** 72-90  **CLD** 80-93 | ^8^ |
| TC, OTC, DOX, SMX, SFD, CIP, ENF | soil | **pH**  5.0  **TOC**  33.24 g kg^-1^  **CEC**  14.40 mol kg^-1^ | EDTA-SPB with ACN:Mg(NO_3_)_2_ NH_3·_H_2_O  (3:1; v/v)  shaken: 30 min, 200 rpm  ultrasonicated 15 min  centrifugation 10 min,  5000 rpm  repart: 3  diluted: 500 mL H_2_O | OASIS HLB  **C:** MeOH, H_2_O  **P:** H_2_O  **E:** 0.1% FA in MeOH  **Ev:** N_2_, 45^o^C | MeOH:0.1% FA in H_2_O  (3:2; v/v) | HPLC-MS/MS | **TC** 104  (SD:11)  **OTC** 95  (SD:5)  **DOX** 106  (SD:13)  **SMX** 111  (SD:5)  **SFD** 77  (SD:10)  **CIP** 62  (SD:11)  **ENF** 89  (SD:14) | ^9^ |
| SMX, SFD, TC, OTC, CIP | sediment | n.d | 0.2 M citric buffer (pH=4):ACN  repart: 3  ultrasonicated 15 min  Ev: rotary evaporator, 55^o^C | OASIS HLB  **C:** MeOH, H_2_O  **P:** H_2_O  **E:** MeOH  **Ev:** N_2_, RT | MeOH: 5 mM oxalic acid, (35:65; v/v) | RRLC-MS/MS | **TC** 48-68  **OTC** 64-72  **CIP** 62-160  **SMX** 69-76  **SFD** 110-123 | ^10^ |
| SMX, ERN, CLR | soil, manure | n.d | Two-steps extraction:  1. ACN  2. EDTA-McIlvaine (pH=4)  ultrasonicated 20 min,  centrifugation 10 min,  5000 rpm  diluted: 200 mL H_2_O, pH=3 | OASIS HLB:  **C:** n.d  **P:** H_2_O  **E:** MeOH  **Ev:** N_2_ | MeOH | LC-MS | manure  79–104  soil  84–116 | ^11^ |
| TC, OTC, DOX, SMX, CIP, ENF | soil, manure | **CEC:**  13.8-25.9  cmol kg^-1^  **OC:**  26.6-31.6  g kg^-1^  **pH:**  4.9-7.6 | EDTA-SPB with ACN:Mg(NO_3_)_2_ NH_3·_H_2_O  (3:1; v/v)  ultrasonicated: 15 min | OASIS HLB:  **C:** n.d  **E:** 0.1% FA w MeOH | - | LC-MS/MS | 77–121 | ^12^ |
| **FQs:**  CIP, ENF  **SAs:**  SMX, SFD  **TCs:**  OTC, DOX | manure | n.d | **FQ:**  AcA:ACN (3:97; v/v)  ultrasonicated: 30 min  centrifugation 10 min,  4000 rpm  repart: 3  **SAs:**  MeOH  shaken: 10 min, 250 rpm, 50^o^C  centrifugation 10 min,  4000 rpm  **TCs:**  1.0% AcA in MeOH  ultrasonicated 30 min,  centrifugation 10 min,  4000 rpm | - |  | HPLC-FLD  HPLC-DAD | **CIP** 92  (RSD: 8)  **ENF** 88  (RSD: 7)  **SMX** 82  (RSD:3)  **SFD** 83  (RSD:3)  **OTC** 64  (RSD:2)  **DOX** 57  (RSD:3) | ^13^ |
| SMX, SFD,  TC, OTC,  CIP, ENF | soil, manure | n.d | ACN:citrate buffer (pH=4)  (1:1; v/v)  ultrasonicated: 15 min  vortex: 1 min  repart: 3  **Ev**: rotary evaporator, 50^o^C  diluted: 100 mL H_2_O | Tandem column:  Waters SAX  OASIS HLB  **C:** MeOH, H_2_O  **P:** H_2_O  **E:** MeOH  **Ev:** N_2_, 50^o^C | MeOH:H_2_O  (1:1; v/v) | UHPLC-MS/MS | soil:  80–105  manure:  71–128 | ^14^ |
| TC, OTC, TYL | soil | n.d | Three-step extraction:  1. citrate buffer (pH=4.2), vortex: 1 min  2. citrate buffer (pH=4.2): ethyl acetate  3. ethyl acetate  Second and third steps:  shaken: 15 min,  centrifugation:  10 min, 1000 rpm  **Ev**: vacuum, 40^o^C | - | ACN:100 mM ammonium acetate  (9:1; v/v) | LC-MS/MS | **TC** 33-46  **OTC** 67-86  **TYL** 60-66 | ^15^ |
| TC, OTC, DOX, CIP | manure | n.d | ACN acidified with formic acid buffer (pH=4.0):0.1 M EDTA-McIlvaine buffer  (1:1; v/v)  centrifugation: 15 min,  4500 rpm  Repart: 2, **Ev:** air  diluted: 100 mL H_2_O | OASIS HLB  **C:** n.d  **E:** MeOH  **Ev**: N_2_, RT | MeOH | HPLC-UV | **TC** 81  (SD/RSD: n.d)  **OTC** 78  (SD/RSD: n.d)  **DOX** 59  (SD/RSD: n.d)  **CIP** 54  (SD/RSD: n.d) | ^16^ |
| OTC, TRI, CIP, ENF | soil, manure | **OC**  0.38-0.89%  **N**  0,016-  0.12%  **pH**  7.1-7.8 | citrate buffer: ACN  ultrasonicated: n.d  diluted: H_2_O + Na_2_EDTA | OASIS Accell  OASIS HLB  **C**: n.d  **E:** MeOH  **P:** H_2_O  **Ev**: N_2_, RT | MeOH:H_2_O  (50:50 v/v) | UHPLC-MS/MS | n.d | ^17^ |
| DOX, TYL, SMX,TRI | manure | n.d | Three-step extraction:  1. 0.125% TFA in ACN  2. 0.125% TFA in ACN: McIlvain-EDTA buffer (pH=4)  (1:1 v/v)  shaken: 15 min  3. (CH_3_COO)_2_Pb, vortex  **e**vaporated: N_2_, 40^o^C  diluted: 13 mL 0.2 M EDTA | Strata-X, Phenomenex  **C:** MeOH, McIlvain-EDTA buffer (pH=4)  **P:** H_2_O  **E:** MeOH  **Ev**: N_2_, 40^o^C | MeOH:H_2_O  (1:4 v/v) | LC-MS | 62-95 | ^18^ |
| SMX  TRI | soil, manure | **OC**  4.0-8.3%  **CEC**  7.6-10.9 cmol/kg  **Clay**  3-24 %  **Silt**  22-39%  **Sand**  37-75%  **pH**  5.58-6.18 | EDTA-McIlvaine (pH=4):MeOH  (1:1; v/v)  shaken: 10 min  ultrasonicated: 40 min  centrifugation: 10 min,  3500 rpm  diluted: 400 mL | Strata-X, Phenomenex  **C:** MeOH, H_2_O  **P:** H_2_O  **E:** MeOH | MeOH | HPLC-MS | **SMX** 60-71  **TRI** 72-80 | ^19^ |
| TC, TYL | soil | n.d | Protease peptone buffer (pH=7)  shaken: 30 min, 4^o^C, 20 rpm  centrifugation: 10 min,  6293 rpm  Repart: 3 | - | - | HPLC-DAD  HPLC-MS/MS | TC, TYL  ~ 100 | ^20^ |
| SMX, TRI | soil | **pH**:  5.5-7.65  **OC:**  38.6-41.5%  **Sand:**  30% | Four-step procedure:  1. 0.1% AcA (R.T, 2 h)  2. 0.1% AcA:ACN:MeOH  (1:4:4; v/v/v)  3. ACN:MeOH (1:1; v/v)  4. 0.5% FA in ACN:MeOH  (1:1; v/v)  ultrasonicated: 20 min  centrifugation: 15 min,  4000 rpm  Ev: N_2_, 24^o^C  Diluted: 100 mL H_2_O | SPE online | - | LC-MS/MS | **SMX**  84  (RSD: 7)  **TRI**  76  (RSD: 1) | ^21^ |
| TRI, SMX, CLD | soil | n.d | 0.1% FA in ACN:H_2_O  (1:1; v/v)  ultrasonicated: 15 min  repart: 3 | - | - | HPLC-HRMS | **SMX** 82-103  **TRI** 85-93  **CLD** 111-135 | ^22^ |
| **SAs:** SMX, SFD, **FQs:** ENF, CIP,  **TCs:** OTC, DOX, TC | manure | n.d | **SAs :**  Na_2_EDTA-McIlvaine  (pH = 4.0):ACN  (7:3; v/v)  ultrasonicated: 20 min  repart: 2  **Ev**: rotary evaporator, 40^o^C  Diluted 40 mL H_2_O | **SAs + FQs:**  OASIS HLB  **C:** MeOH: H_2_O  **E**: MeOH  **Ev**: N_2_, 40^o^C | MeOH:H_2_O  (1:1 v/v) | HPLC-MS/MS | n.d | ^23^ |
|  |  |  | **FQs:**  Na_2_EDTA-McIlvaine  (pH=7.0):ACN  (1:1; v/v)  ultrasonicated: 20 min  repart: 2  **Ev**: rotary evaporator, 40^o^C  Diluted 40 mL H_2_O |  |  |  |  |  |
|  |  |  | **TCs:**  ACN: phosphate buffer (pH=3)  (1:1; v/v)  shaken: 10 min  ultrasonicated: 20 min  centrifugation: 5 min,  7000 rpm  Diluted: 130 mL H_2_O,  pH=2,8-3,0 | **TCs:**  Tandem column:  Waters SAX  OASIS HLB  **C:** MeOH, H_2_O, phosphate buffer (pH=3)  **P:** H_2_O, 5% MeOH  **E:** MeOH  **Ev:** N_2_, 40^o^C |  |  |  |  |
| SMX, SFD, TYL, OTC, TC, DOX, CIP, ENF | sediment manure | n.d | citrate acid buffer (pH=3):ACN  ultrasonicated: 15 min  centrifugation: 10 min  Ev: rotary evaporator, 50^o^C  Diluted 200 mL H_2_O, pH=3 | Tandem column:  Waters SAX  OASIS HLB  **C:** MeOH, H_2_O  **P:** H_2_O  **E:** MeOH  **Ev:** air | 0.2% FA in MeOH: 2 mM ammonium acetate  (30:70 v/v) | RRLC-Ms/MS | **SFD** 55-59  **SMX** 85-112  **TYL** 103-137  **OTC** 44-94  **TC** 44-94  **DOX** 73-155  **CIP** 80-88  **ENF** 231-325 | ^24^ |
| DOX, OTC, SFD, TRI, TYL | manure | n.d | 6% TCA in ACN  shaken: 30 min  centrifugation: 15 min  Ev: N_2_, 45^o^C | - | H_2_O:ACN (80:20 v/v) | UHPLC-MS/MS | **DOX** 98-100  **OTC** 94-98  **SFD** 104-107  **TRI** 98-103  **TYL** 103-118 | ^25^ |
| TC, OTC, DOX, TRI, TYL | soil | n.d | EDTA-McIlvaine buffer (pH=5)  ultrasonicated: 15 min, 25^o^C  centrifugation: 15 min,  6000 rpm | Strata-X, Phenomenex  **C:** MeOH, H_2_O, McIlvaine buffer (pH=5)  **P** MeOH:H_2_O  (5:95 v/v)  **E:** 0.1% AcA in MeOH  **Ev**: N_2_, RT | H_2_O | LC-HMRS | 40-111 | ^26^ |
| ENF,  CIP | soil | **pH:**  6.6-7.7  **OC**  1.8-7.2 %  **SiO_2_**  43.1-79.2  **Sand**  24.5-75.7%  **Slit**  13.3-53.1%  **Clay**  11.0-28.1% | ACN: phosphate buffer (pH=12)  (75:25 v/v)  or  acetone phosphate buffer (pH=12)  (60:40 v/v)  shake: 90 min  centrifugation: 20 min, 3500 rpm  pH=4 | Bond Elute C18  **C:** MeOH, H_2_O, phosphoric buffer (pH=7)  P: H_2_O  **E:** 1.0M NH_3_ in ACN  **Ev:** N_2_, 40^o^C | Oxalic acid buffer (pH=4):ACN  (88:12 v/v) | LC-FLD | **CIP** 42-48  **ENF** 62-70 | ^27^ |
| CLR, TRI, SMX, SFD | soil | n.d | Two-step extraction:  1. 0.1% FA in ACN:H_2_O  (1:1; v/v)  2. 0.1 % FA in  ACN:2-propanol:H_2_O  (3:3:4; v/v/v)  ultrasonicated: 15 min | - | - | LC-MS/MS | **CLR** 112-126  **TRI** 92-114  **SMX** 101-113  **SFD** 73-82 | ^28^ |

TC – tetracycline, DOX – doxycycline, OTC – oxytetracycline, TYL – tylosin, SMX – sulfamethoxazole, SFD – sulfadiazine, TRI – trimethoprim, CLR – clarithromycin, CLD – clindamycin, CIP – ciprofloxacin, ENF – enrofloxacin, CEC – cation exchange capacity, OC – organic carbon, TOC – total organic carbon, SPB – sodium phosphate buffer,

ACN – acetonitrile, FA – formic acid, AcA – acetic acid, FLD – fluorescence detector, DAD – diode array detector, RT – room temperature, Ev – evaporated, TCs – tetracyclines, SAs – sulphonamides, FQs – fluoroquinolones, n.d – no data, C – conditioning, E – elution, P – purification, LC – liquid chromatography, HPLC – high-performance liquid chromatography, UHPLC – ultra-high-performance liquid chromatography, MS/MS – tandem mass spectrometer, HRMS – high-resolution mass spectrometry, RRLC – rapid resolution liquid chromatography, MCAC – metal chelate affinity chromatography, SD – standard deviation, RSD – relative standard deviation

**Table S2.** MRM (multiple reaction monitoring) parameters of the antimicrobials analyzed in this study

| **Analyte** | **Q1 (m/z)** | **Q3 (m/z)** | **DP^a^ (V)** | **CE^b^ (V)** | **EP^c^ (V)** | **CXP^d^ (V)** |
| --- | --- | --- | --- | --- | --- | --- |
| CIP | 332.0 | 288.0  314.0 | 86 | 25  29 | 25  29 | 10  8 |
| CLD | 425.6 | 126.2  127.2 | 166 | 43  39 | 43  39 | 10  8 |
| CLR | 748.4 | 590.0  558.4 | 136 | 27  31 | 27  31 | 8  8 |
| DOX | 445.2 | 428.2  154.2 | 111 | 27  41 | 27  41 | 12  8 |
| ENF | 360.9 | 316.2  245.1 | 101 | 29  27 | 29  27 | 8  10 |
| LVF | 361.9 | 318.2  261.2 | 221 | 27  37 | 27  37 | 10  6 |
| MET | 172.1 | 128.1  82.1 | 76 | 21  33 | 21  33 | 8  6 |
| OTC | 461.1 | 426.0  443.0 | 106 | 29  17 | 29  17 | 20  14 |
| SFD | 250.8 | 108.1  156.2 | 71 | 33  23 | 33  23 | 8  12 |
| SMX | 253.9 | 92.1  108.1 | 96 | 43  37 | 43  37 | 6  8 |
| TC | 445.1 | 410.2  427.2 | 106 | 27  19 | 27  19 | 12  18 |
| TRI | 290.9 | 230.2  261.2 | 116 | 33  35 | 33  35 | 6  6 |
| TYL | 916.1 | 174.2  775.5 | 161 | 53  41 | 53  41 | 14  12 |
| VAN | 725.6 | 144.1  256.2 | 76 | 23  31 | 23  31 | 10  14 |

^a^ declustering potential

^b^ collision energy

^c^ entrance potential

^d^ collision cell exit potential

**Table S3.** Selected physical and chemical parameters of various matrices used in the experiment

| **Sample** | **Dry matter^a^ (g g^-1^)** | **Water content^a^ %** | **Organic matter^b^ (g g^-1^)** |
| --- | --- | --- | --- |
| Soil | 0.885±0.001 | 11.5% | 0.015±0.000 |
| Sand | 0.9996±0.000 | 0.04% | 0.000 |
| Chicken manure | 0.257±0.006 | 74.3% | 0.202±0.005 |
| Cattle manure | 0.415±0.018 | 58.5% | 0.129±0.000 |

^a^ determined using gravimetric method, according to ISO 11465:1993,

^b^ determined using the dry combustion method, according to ISO 10694:1995.

## S4. Supplementation to section 3.1 Step by step to the effective extraction procedure – development of the SLE-SPE conditions for extraction of AMs from soil samples

***S4.1 SLE procedure***

***a) Selection of solvent and time***

Mixtures of citrate buffer (pH=4) with an organic solvent in a 1:1 volume ratio were good extractants for 7 ~~out~~ of the 14 selected AMs (TYL, TRI, MET, CLR, CLD, SMX, SFD). Citrate buffer (pH=4) in a mixture of acetonitrile extracted seven mentioned above AMs with an efficiency of over 85% (88% for SMX – 110% for CLD). A mixture of citrate buffer (pH=4) with methanol for the same compounds gave a very similar recovery for the same analytes (78% for TRI – 98% for SFD). The use of a citrate buffer at pH=2 in a mixture with acetonitrile gave a 20 – 30% lower recovery than in the procedure where the buffer at pH=4 was used. A similar extraction yield for the same seven analytes was obtained using the mixture of methanol:ethyl acetate (1:1; v/v); 68% SMX – 104% MET. The obtained results indicate that none of the listed extraction mixtures was an effective extractant for TCs (26–32%) and FQs (5–16%). A reduction in the extraction efficiency was observed under acidic conditions such as 0.1% formic in methanol and mixtures of acetonitrile with 0.1% formic acid in water. Moreover, under acidic conditions, no signals from TC, OTC, or DOX were observed in the chromatograms, and the recovery of FQs was about 12%. Other results were obtained under basic conditions. The use of 2% ammonia in methanol did not allow efficient analyte extraction, and the recovery ranged from 4% (ENF) to 94% (TRI); however, the use of 0.2 M sodium hydroxide in water for extraction increased the extraction efficiency of FQs, which ranged from 30% (LVF) to 53% (ENF).

Combining McIlvaine buffer (pH=4) with methanol resulted in the extraction of 45% TCs and 75% VAN. This mixture was also appropriate for extraction of TYL, TRI, MET, CLR, and CLD (similar that using citrate buffer (pH=4):methanol (1:1; v/v)). Therefore it was selected as one of the extraction solvents. In the subsequent studies, the highest recovery of TCs was obtained using a mixture of McIlvaine buffer (pH=4):acetonitrile (1:1; v/v); 80% OTC and 87% TC therefore, this mixture was selected as a second solvent used for SLE procedure.

Only one solvent, a mixture of 0.2 M NaOH in water and acetone (1:1; v/v), successfully extracted the FQs and SAs. It is in line with the literature where a two-step extraction was used to extract six FQs, where a 0.1 M sodium hydroxide solution was used in the second step ^8^. A mixture of organic solvent:0.2 M sodium hydroxide (1:1; v/v) was used to improve the extraction efficiency. The acetone-containing mixture had the highest recovery of FQs and SAs (85–107%). Similar results were obtained for a mixture of 0.2 M sodium hydroxide with acetonitrile (79–97%) and methanol (75–86%, except LVF 55%). Finally, a mixture of 0.2 M sodium hydroxide:acetone (1:1; v/v) was selected as a third solvent used in the extraction procedure.

It was found that a time of 30 min was the maximum time for a single extraction using buffered solutions (Figure S1). After 30 min, the AMs recovery was in the range of 82–111% for citrate buffer (pH=4):acetonitrile (1:1; v/v) and 80–108% for McIlvaine buffer:methanol (1:1; v/v). After 60 min of agitation in citrate buffer (pH=4):acetonitrile (1:1; v/v) significant losses were observed, and the recoveries were 50% (VAN), 41% (CLR), 63% (SMX), and 78% (SFD). There was also a 5–20% loss for the remaining analytes compared with shorter shaking times. During the 30 min extraction with 0.2 M sodium hydroxide:acetone (1:1; v/v), analyte losses of 10–34% were observed, and at 60 min, the losses were higher (7–50%). As expected, FQs were stable under alkaline conditions, and the recoveries were 70–93% for CIP, 73–110% for LVF, and 69–89% for ENF. It was assumed that this solvent would be suitable for extracting FQs from samples in the next stages, as long as the maximum shaking time was 30 min.

**
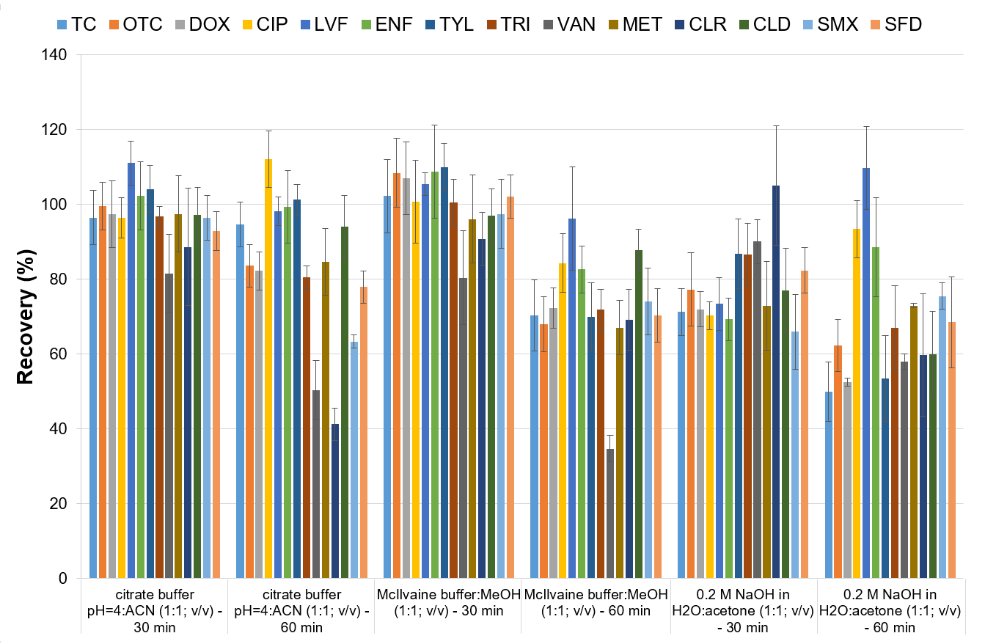
**

**Figure S1.** Stability of the analytes in the solvent during different times of shaking (30 min and 60 min)

***b) Application of ultrasound-assigned extraction***

As the next step, it was checked whether using ultrasound-assisted extraction (UAE) would increase the procedure's effectiveness. The results were obtained by comparing the samples after UAE and without UAE. The obtained results are presented in Figure S2. UAE promotes the desorption of analytes from the soil matrix. The time of single extraction usually is from 10 to 30 minutes (Table S1). The UAE application decreased analytes' recovery to between 20% and 60%. The highest decreases were observed for CIP (59%), LVF (59%), and CLD (31%). These losses were considered too high for the selected group of analytes, and UAE was abandoned. Using ultrasound for more than 10 min lowers the AMs recoveries ^19,27^. One reference indicated that extending the UAE time did not increase the recovery of analytes because the extraction mechanism was controlled by the distribution coefficient ^29^. In our opinion, this may also be related to the instability of AMs toward ultrasounds.

**
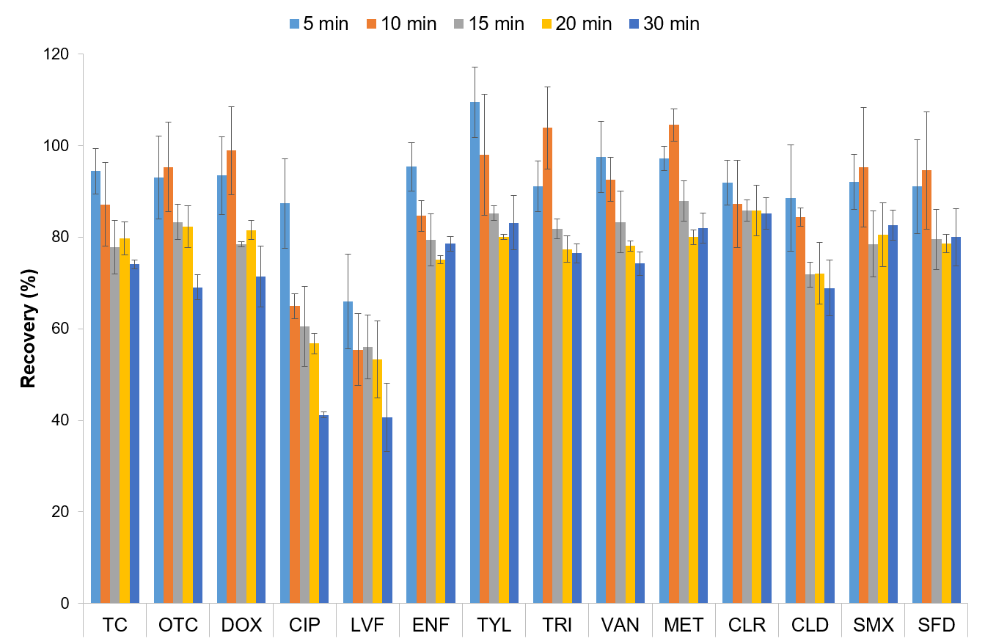
**

**Figure S2.** Effect of UAE on the extraction efficiency (5–30 min; extraction mixture: citrate buffer (pH=4):acetonitrile (1:1; v/v))

***S4.2 SPE procedure***

For soil samples, the matrix composition significantly impacted the SPE efficiency. Co-extraction of soil matrix components such as, e.g., humic and fulvic acids, reduced the recovery during the SPE stage ^23,24^. Most often, cartridges are combined in tandem to remove matrix components and retain AMs 3,5 simultaneously. To verify the effect of the matrix on the SPE efficiency, (a) tests were carried out on an enriched matrix extract, (b) on a sample containing no organic matter, i.e., quartz sand, and (b) columns were connected in tandem.

***a) Selection of solvent used to dilution extract before SPE***

After SLE, the sample was filtered, and the extract was purified using SPE. The obtained extract was a mixture of water and organic solvent, so it should be evaporated or diluted in water before SPE. The evaporation of mixtures of water:organic solvents were long (> 8 h), caused a significant loss of the analytes, which finally gave a low recovery of TC (6.0–26.7%) and FQs (5–14.9%).

A better solution was to dilute the extract to obtain an organic solvent content lower than 5%. In this case, a higher analyte recovery was obtained for TCs (46.7–74.3%), FQs (29.8– 83.9%), and VAN (64.6–92.4%). The recoveries for the remaining AMs (SMX, SFD, TYL, TRI, CLR, and CLD) were comparable to those obtained after evaporation. Finally, it was decided that an evaporation step of the SLE extract (water:organic solvent) was inadvisable, and the extracts (McIlvaine buffer (pH=4):methanol (1:1; v/v); McIlvaine buffer (pH=4): acetonitrile (1:1; v/v) and 0.2 M sodium hydroxide solution:acetone (1:1; v/v)) obtained after SLE were diluted with water adjusted to pH=3 before SPE.

***b) The influence of the soil matrix on the efficiency of the SPE procedure***

In the next step, the effect of the sample matrix on the SPE efficiency was studied, so the SPE efficiency for analytes dissolved in the pure solvent and for analytes dissolved in matrix extracts (after SLE) was compared. Soil extracts must be cleaned before LC-MS/MS analysis to remove co-extracted salts, humic and fulvic acids, the main constituents of soil organic matter, which could deposit on the sorbent instead or may interfere in the ionization of the target analytes ^21^. It was noticed that the components of the soil matrix had a significant influence on the pH of the solution. Information on selected soil parameters is presented in Table S3. The pH values of diluted solvents with added AMs were pH=4.2 for citrate buffer (pH=4) with acetonitrile and pH=11.1 for 0.2 M sodium hydroxide: acetone, while those of the obtained matrix solutions were respectively pH=6.2 and pH=8.1. The results of SPE are shown in Figure S3. For samples containing no matrix components (solution 1, solution 2), the recovery of the individual groups of AMs was as follows: TCs (46.7–101%), SAs (88.7–106%), FQs (41.0–92.4%), macrolides (91.1–109%), TRI (97.3–103%), MET (40.5–50.3%) VAN (63.7–93.2%), and CLD (81.3–85.6%). In the samples containing the soil matrix (soil extract 1 and 2), the recovery was much lower: TCs (6.6–17.3%), SAs (30.4–36.5%), FQs (17.4–42.8%), macrolides (54.9–75.6%), TRI (64.8–71.4%), MET (33.6–44.4%) VAN (50.6–63.3%), and CLD (48.8–51.1%). These results show that co-extracting matrix compounds and AMs significantly reduced the SPE efficiency. This suggests that an additional purification step should be used to remove matrix components that reduce the analyte recovery.

In relation to the above results, the influence of soil organic matter studies was performed using two types of samples: quartz sand and soil. The organic matter content in the soil samples was at the level of 0.015 g/g, while the sand did not contain any organic matter (Table S3). The content of organic matter in soil is correlated with the content of soil organic carbon ^30^, and this parameter (depending on the physicochemical properties of the tested compound) is mentioned as one of the factors influencing the affinity of a given substance to the matrix ^31^. The recovery of the analytes from the enriched quartz sand samples was higher than ~~for~~ the enriched soil samples using the same extraction procedure. It is due to two factors: the sorption of analytes into the organic matrix and the co-elution of matrix components that deactivated the SPE sorbent. The results are summarized in Figure S4. For extracted soil samples, the loss of analytes ranged from 60.9–100%. No DOX signals were observed in the chromatogram of soil extracts, while for TCs and FQs, the maximum recovery was about 18%. The highest recovery was obtained in McIlvaine buffer (pH=4):methanol for CLD (35%) and TYL (34%).


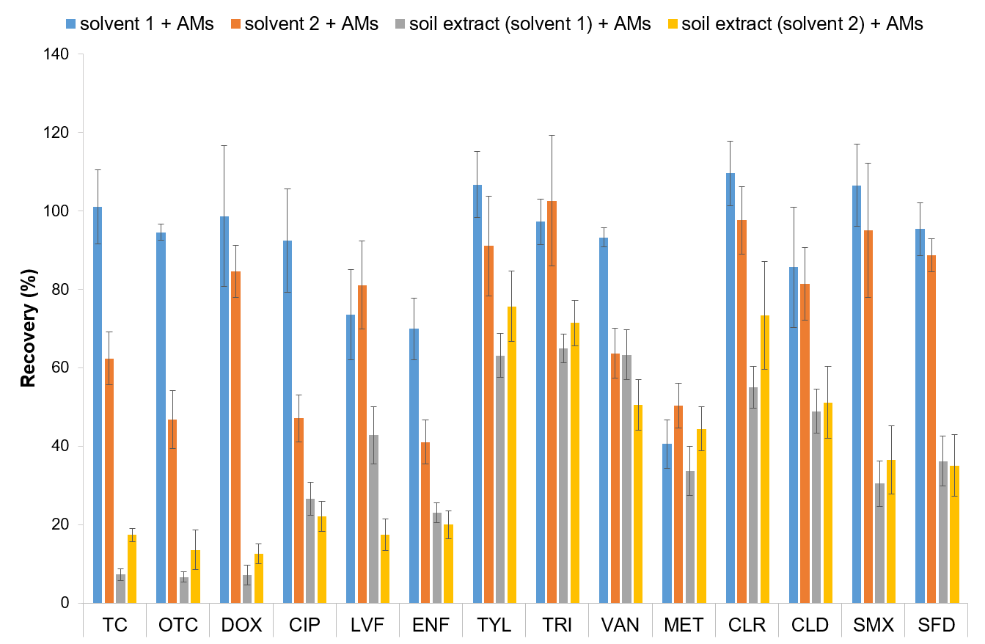


**Figure S3.** Influence of matrix (comparison of pure solvent and soil extract) on the efficiency of the SPE procedure (solvent 1: citrate buffer (pH=4):acetonitrile (1:1; v/v);
solvent 2: 0.2 M sodium hydroxide:acetone (1:1; v/v)

***
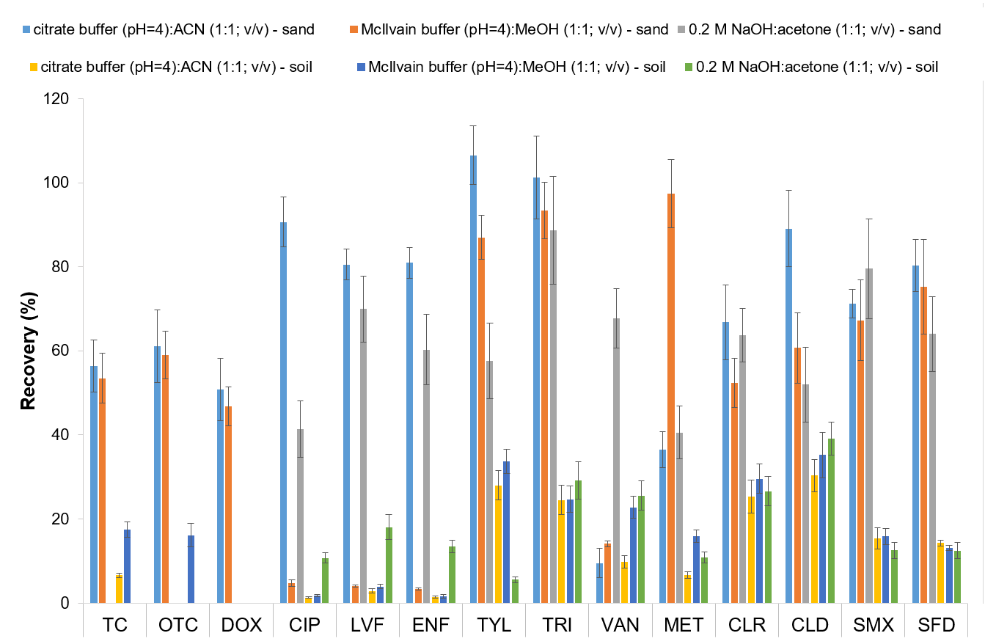
***

**Figure S4.** Effect of type of matrix (soil samples and quartz sand) on the efficiency of the SPE procedure

Each analyte recovery was 50% or greater in the quartz sand extracts. Buffered solutions such as citrate buffer (pH=4):acetonitrile and McIlvaine buffer (pH=4):methanol gave similar recovery values for TCs, in the range 47–61%. The mixture of acetonitrile with citrate buffer at pH=4 was the best extractant for FQs (80–106%), TYL (106%), TRI (101%), and CLR (67%), CLD (89%), and SFD (80%). McIlvaine buffer with methanol had a MET extraction efficiency of 97%, while a solution of 0.2 M sodium hydroxide with acetone had the highest VAN extraction efficiency (68%). It was shown that the SPE step significantly reduced the extraction efficiency of analytes from soil samples. Table S1 shows that the basic soil parameters (sand, silt, clay, and organic carbon) are not given for each procedure. An additional soil cleaning step was ~~most~~ often necessary for clay and silt ^4,6^. Soils with a sand content close to 70% did not require the extract to be cleaned before SPE ^19,27^. This experiment also confirmed that it is necessary to introduce an additional sample purification step before SPE.

***c) Selection of tandem-SPE conditions***

As the above studies showed, an additional step to remove interferents was applied in the SPE procedure. For this reason, tandem SPE was applied, in which the impurities were retained on the first SPE column while the analyte remained in the liquid phase and was then retained in the second SPE column. The use of an additional SPE cartridge on top of the HLB is a common method to reduce matrix interference by adsorbing anionic humic particles from soil extracts ^6,24^. First, three types of cartridges with different sorbents were tested, and the results are presented in Table S4. OASIS WAX was the best as a first column (for removing interferences, not retaining analytes) because it did not retain 9 of the 14 analytes. After eluting analytes from the OASIS WAX cartridge with methanol, small amounts of CIP (4.7%), MET (1.4%), CLR (12.4%), SMX (27.7%), and SFD (12.0%) were detected in the eluate. The analytes were better adsorbed in the other two types of sorbents, which is undesirable. The Bakerbond C18 cartridge retained 8 of the 14 analytes (recoveries from 4.4% for SFD to 84.9% for TRI), and the Bond Elut Plexa retained 11 of the 14 analytes (recoveries from 5.7% for ENF to 93.3% for TRI).

In the next step, the selected OASIS WAX column was connected with an OASIS HLB column, and the soil extract enriched with AMs was passed through them. After elution, the methanol eluates were collected individually from each of the SPE columns. As predicted, in the OASIS HLB eluate, the recovery of AMs ranged from 8.7% (SMX) to 116% (MET), and for 12 of the 14 analytes, it was above 50%. The lowest recovery was for the sulfonamide AMs SMX (8.7–9.9%) and SFD (37.5–53.8%). OASIS WAX retained SFD (27.4%), SMX (56.3%), CLR (27.6–42.1%), and TYL (15.0–36.5%). The recovery of the remaining 10 AMs was below 23% using only the OASIS WAX cartridge. According to the literature, after SPE, the pre-column was removed, and only the HLB cartridge analytes were eluted ^3,5^. In our research, the eluates obtained from both the OASIS WAX and OASIS HLB cartridges were clear, which means that the matrix components were adsorbed on the OASIS WAX column and were not desorbed during elution. Therefore, combining the eluents obtained from both SPE columns was decided to obtain the highest analyte recovery.

The last stage of the experiment was the selection of the pH of the extract applied to the SPE column and the composition and volume of the eluate. From the procedures listed in Table S1, the soil extract was adjusted to a pH range of 2.3 – 4.0, which corresponds to the pH at which the analytes were ionized and bonded more easily to the SPE sorbent. For the OASIS HLB cartridge, the samples were typically adjusted to pH 3 ^5,11,24^, or 4 ^3,27^, so these two values were investigated. The lowest analyte recoveries (39.1–87.5%) were obtained where the sample extract pH was 4, and elution was performed with pure methanol (procedure B). Sequential elution with 0.1% acetic acid in methanol slightly increased the recovery compared with pure methanol (47–96.9%, procedure C). Lowering the pH of the sample extract to pH 3 gave better recovery for the same analytes (procedure A) than pH 4 (procedure B) when methanol was used as the eluent. It was decided that adjusting the soil extract to pH 3 would give the highest analyte recovery. In the next step, two types of eluents were investigated – the columns were eluted sequentially with pure methanol and 0.1% ammonia in methanol (procedure D) and pure methanol and 0.1% acetic acid in methanol (procedure E). Procedures D and E gave similar recoveries for TCs, TRI, MET CLD, and SAs, but procedure E gave a higher recovery of FQs and CLR. The final optimized extraction conditions for SPE are provided in Section 2.3.1.

**Table S4.** Recovery of analytes using various types of SPE (solid-phase extraction) cartridges

| SPE parameters | | | | Recovery (%) (SD (%)) | | | | | | | | | | | | | |
| --- | --- | --- | --- | --- | --- | --- | --- | --- | --- | --- | --- | --- | --- | --- | --- | --- | --- |
| Column | SLE solvent | pH (acid) | Elution (volume [mL]) | TC | OTC | DOX | CIP | LVF | ENF | TYL | TRI | VAN | MET | CLR | CLD | SMX | SFD |
| WAX  (60 mg,  3 mL) | 0.2 M NaOH: acetone | 4.0 (FA) | MeOH  (6) | - | - | - | 4.7  (0.4) | - | - | - | - |  | 1.4  (0.4) | 12.4  (1.4) | - | 27.7  (1.6) | 12.0  (1.1) |
| C18  (60 mg,  3 mL) | 0.2 M NaOH: acetone | 4.0 (FA) | MeOH  (6) | - | - | - | - | - | - | 55.6  (8.7) | 84.9  (11.8) | 22.7  (2.4) | 5.7  (0.87) | 43.0  (6.5) | 14.0  (1.0) | 25.0  (1.9) | 4.4  (1.0) |
| Plexa  (60 mg,  3 mL) | 0.2 M NaOH: acetone | 4.0 (FA) | MeOH  (6) | - | - | - | 6.7  (1.1) | 10.5  (1.1) | 5.7  (0.7) | 49.1  (9.2) | 93.3  (3.9) | 31.8  (5.4) | 6.0  (0.8) | 48.7  (4.2) | 63.3  (9.7) | 32.3  (2.3) | 22.3  (0.6) |
| WAX  (60 mg,  3 mL) | citrate buffer (pH=4):ACN (1:1; v/v) | 4.0 (FA) | MeOH:0.1% AcA w MeOH  (3:3) | 14.6  (2.3) | 11.1  (0.4) | 19.8  (3.1) | 17.2  (2.2) | 12.3  (1.6) | 22.8  (3.0) | 15.0  (2.0) | 13.9  (1.2) | - | 5.7  (0.4) | 27.6  (5.6) | 11.5  (1.5) | 45.8  (3.7) | 36.5  (3.0) |
| HLB  (500 mg,  6 mL) |  |  | MeOH:0.1% AcA w MeOH  (6:6) | 58.6  (13.1) | 63.4  (1.8) | 71.7  (11.8) | 61.8  (5.7) | 90.3  (6.5) | 74.0  (9.2) | 92.0  (13.9) | 94.8  (5.8) | 77.3  (6.1) | 116.3  (9.1) | 74.2  (9.3) | 78.4  (6.9) | 8.7  (1.1) | 37.5  (3.3) |
| WAX  (60 mg,  3 mL) | McIlvaine buffer (pH=4):MeOH (1:1; v/v) | 4.0 (FA) | MeOH:0.1% AcA  w MeOH  (3:3) | 3.0  (0.4) | 6.11  (1.02) | 4.2  (0.9) | 10.6  (0.5) | 6.8  (0.2) | 14.5  (1.9) | 36.5  (6.7) | 6.3  (0.7) | - | 1.9  (0.1) | 42.1  (3.9) | 5.4  (1.9) | 56.4  (2.5) | 27.4  (5.9) |
| HLB  (500 mg,  6 mL) |  |  | MeOH:0.1% AcA w MeOH  (6:6) | 46.6  (8.4) | 40.2  (8.2) | 52.9  (11.9) | 27.3  (4.2) | 49.9  (5.9) | 25.6  (4.6) | 73.3  (11.0) | 84.3  (4.5) | 68.7  (6.7) | 61.1  (2.8) | 58.2  (4.2) | 67.0  (8.8) | 9.9  (0.8) | 53.8  (3.8) |

***S4.3 evaporation, dissolving, and filtration***

***a) Evaporation of the extract***

Based on the conducted studies, it can be concluded that the sample evaporation stage was a critical stage that affected the repeatability of the obtained results. Thus, samples were evaporated with flowing nitrogen due to the high recovery of the analyte 82.0% (DOX) – 103% (CIP), short evaporation time, and high reproducibility. As expected, evaporation under the nitrogen stream increased the recovery of AMs by approx. 5–10% (TCs, TYL, TRI, MET, SMX, CLD, CLR). In the case of samples evaporated with air, the evaporation time was from 8 – 12 h, which influenced the reproducibility, and the recoveries ranged from 56.6% (CLR) to 104% (CLD). Figure S5 presents the results obtained after evaporating the extract with nitrogen and air. In this work, evaporation was conducted at room temperature due to a large number of analytes with different chemical and thermal stabilities ^32^.

***
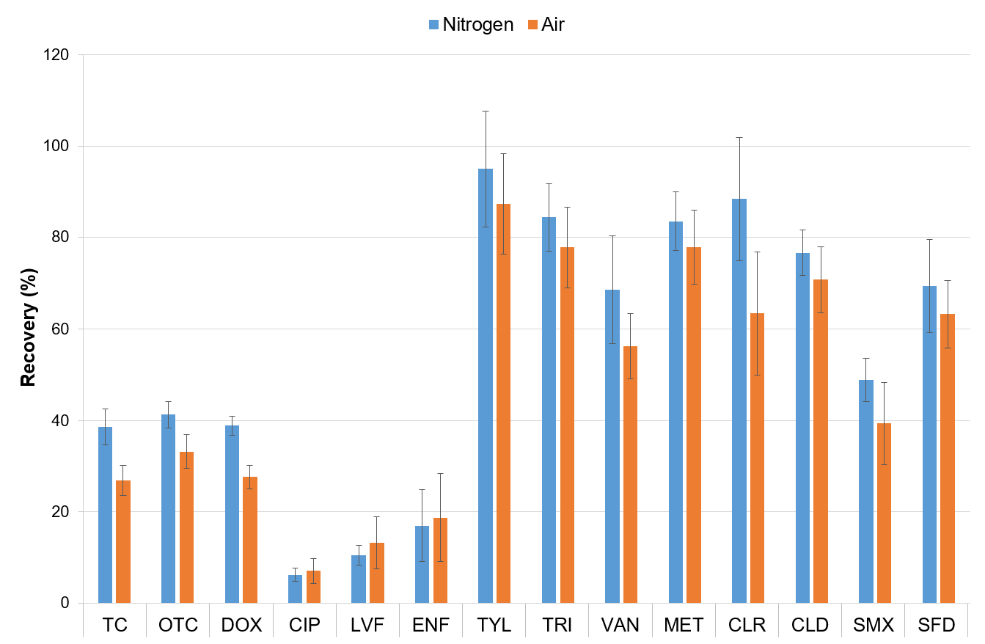
***

**Figure S5.** Effect of evaporation conditions on the recovery of AMs

***b) Dissolving residues***

Five solvents with different polarities were selected to dissolve the residues obtained after eluate evaporation prior to LC-MS/MS analysis, and the results are shown in Figure S6. Dissolving the sample in acetonitrile gave a low recovery for TCs (<2%), SAs (<29%), and FQs (<5%), and only TRI, CLD, and MET had recoveries above 60%. Acidification of acetonitrile slightly increased the recovery for 10 of 14 analytes, but it was lower than 72%. For methanol, which is most commonly used in the literature ^11,16,19^, the recovery of analytes was in the range of 41.1 – 112%, while for TC, DOX, OTC, and CLD, it was less than 60%. The use of a mixture of 0.1% FA in water:acetonitrile (9:1; v/v) as a solvent gave a recovery of <50% for 8 out of 14 AMs. According to the literature, data methanol was also used in a mixture with water in various volume ratios to dissolve the residues containing AMs ^3,14,17^. The highest analyte recovery (85.2–106%) was obtained for a mixture of 0.1% formic acid in water: methanol (1:1; v/v). This result is in line with the literature, where using acidified water in a mixture with methanol resulted in the highest analyte recovery during the reconstituted stage ^7–9^.


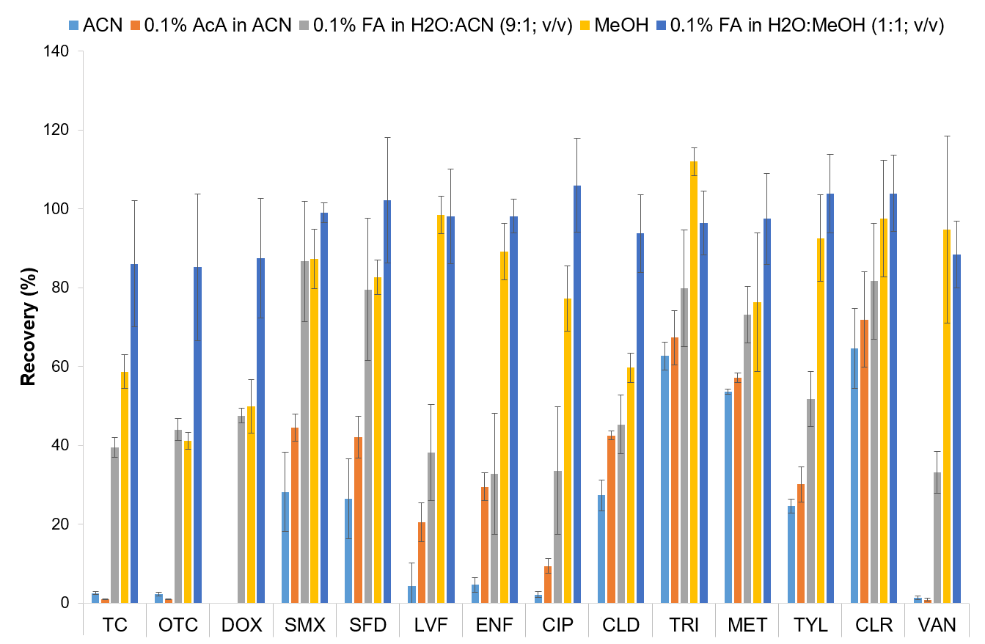


**Figure S6.** Effect of the solvents used for the dissolved of residues after SLE-SPE procedure on the recovery of analytes

***c)*** ***Filtration of the extract***

A syringe filtration step prior to LC-MS/MS analysis was necessary due to the turbidity of the soil extract solution. The filtration efficiency of the analytes was checked. Nylon and polyethersulfone syringe filters with different sizes and porosities were tested. The syringe filters were selected based on our previous reports about AMs ^33,34^. The highest recoveries of 81.7% (CLR) and 119% (OTC) were obtained using Nylon 66 syringe filters (0.45 µm, Ø25 mm). PES syringe filters (0.45 µm, Ø13 mm) also gave good recoveries in the range of 70.2% (CLD) to 102% (DOX), but these were worse for some analytes than using nylon filters (Figure S7). Finally, the extract was then filtered through a nylon syringe filter.


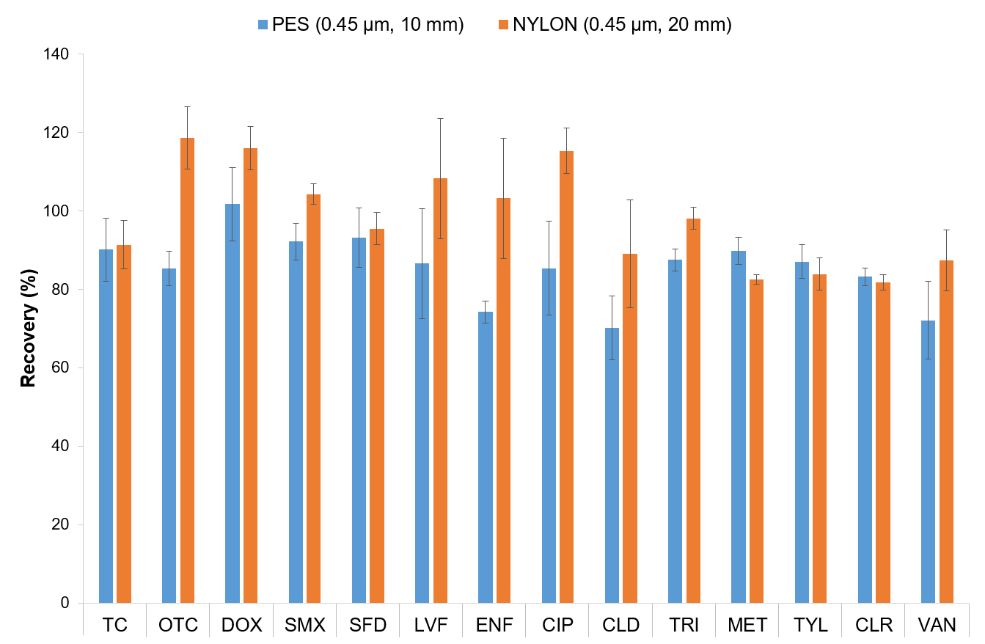


**Figure S7.** Effect of the filter materials on the recovery of analytes

# *S5. Supplementation to section S5. Method validation*

The LC-MS/MS method applied conditions were validated on extracts obtained from blank samples (soil samples without selected AMs), which were enriched with an appropriate amount of the standard solution. The analyses were performed each time for three parallel samples in duplicate (six independent repetitions), and the results are shown in Table 2. The calibration curves for the 14 selected AMs were linear over the concentration range from 0.5 ng g^-1^ to 500.0 ng g^-1^. The equations of the curves were determined using the linear regression method, and the obtained coefficient of determination (*R*^2^) was higher than 0.99.

The sensitivity of the method was determined by the LOD and LOQ parameters, which were 0.17 ng g^-1^ and 0.5 ng g^-1^, respectively. The selectivity was determined by comparing two chromatograms – a soil matrix enriched with AMs and a pure matrix (blank). No interference from matrix components was observed in the chromatogram. The analyte recovery for the two developed SLE-SPE procedures was determined. Soil samples were enriched with analytes at three concentration levels (LQC, MQC, and HQC), and extractions were performed. The recovery of the analytes ranged from 60–95% in procedure 1 and 70–96% in procedure 2 in HQC, which are satisfactory. Precision, defined as the coefficient of variation (CV) for all analytes, was less than 7.89%. The accuracy was determined as a relative error (RE) between -6.35% and 6.54% for all analytes. The matrix effect was also determined, but for the analytes, it was negligible (ME=10.5%).

# Reference

1. McIlvaine, C. A buffer solution for colorimetric comparsion. *J. Biol. Chem.* **49**, 183–186 (1921).

2. De Liguoro, M., Cibin, V., Capolongo, F., Halling-Sørensen, B. & Montesissa, C. Use of oxytetracycline and tylosin in intensive calf farming: Evaluation of transfer to manure and soil. *Chemosphere* **52**, 203–212 (2003).

3. Hou, J. *et al.* Occurrence and distribution of sulfonamides, tetracyclines, quinolones, macrolides, and nitrofurans in livestock manure and amended soils of Northern China. *Environ. Sci. Pollut. Res.* **22**, 4545–4554 (2015).

4. Yi, X., Bayen, S., Kelly, B. C., Li, X. & Zhou, Z. Improved detection of multiple environmental antibiotics through an optimized sample extraction strategy in liquid chromatography-mass spectrometry analysis. *Anal. Bioanal. Chem.* **407**, 9071–9083 (2015).

5. Blackwell, P. A. *et al.* Ultrasonic extraction of veterinary antibiotics from soils and pig slurry with SPE clean-up and LC-UV and fluorescence detection. *Talanta* **64**, 1058–1064 (2004).

6. Hu, W. *et al.* Simultaneous extraction and determination of fluoroquinolones, tetracyclines and sulfonamides antibiotics in soils using optimised solid phase extraction chromatography-tandem mass spectrometry. *Int. J. Environ. Anal. Chem.* **92**, 698–713 (2012).

7. Ho, Y. B., Zakaria, M. P., Latif, P. A. & Saari, N. Simultaneous determination of veterinary antibiotics and hormone in broiler manure, soil and manure compost by liquid chromatography-tandem mass spectrometry. *J. Chromatogr. A* **1262**, 160–168 (2012).

8. Bian, K. *et al.* Determination of multi-class antimicrobial residues in soil by liquid chromatography-tandem mass spectrometry. *RSC Adv.* **5**, 27584–27593 (2015).

9. Huang, Y. *et al.* Simultaneous extraction of four classes of antibiotics in soil, manure and sewage sludge and analysis by liquid chromatography-tandem mass spectrometry with the isotope-labelled internal standard method. *Anal. Methods* **5**, 3721–3731 (2013).

10. Yang, J. F. *et al.* Simultaneous determination of four classes of antibiotics in sediments of the Pearl Rivers using RRLC-MS/MS. *Sci. Total Environ.* **408**, 3424–3432 (2010).

11. Gu, D. *et al.* Occurrence and Risk Assessment of Antibiotics in Manure, Soil, Wastewater, Groundwater from Livestock and Poultry Farms in Xuzhou, China. *Bull. Environ. Contam. Toxicol.* **103**, 590–596 (2019).

12. Zhang, H. *et al.* Residues and risks of veterinary antibiotics in protected vegetable soils following application of different manures. *Chemosphere* **152**, 229–237 (2016).

13. Zhao, L., Dong, Y. H. & Wang, H. Residues of veterinary antibiotics in manures from feedlot livestock in eight provinces of China. *Sci. Total Environ.* **408**, 1069–1075 (2010).

14. Li, C. *et al.* Occurrence of antibiotics in soils and manures from greenhouse vegetable production bases of Beijing, China and an associated risk assessment. *Sci. Total Environ.* **521**–**522**, 101–107 (2015).

15. Hamscher, G., Sczesny, S., Höper, H. & Nau, H. Determination of persistent tetracycline residues in soil fertilized with liquid manure by high-performance liquid chromatography with electrospray ionization tandem mass spectrometry. *Anal. Chem.* **74**, 1509–1518 (2002).

16. Hu, X. G., Luo, Y., Zhou, Q. X. & Xu, L. Determination of thirteen antibiotics residues in manure by solid phase extraction and high performance liquid chromatography. *Fenxi Huaxue/ Chinese J. Anal. Chem.* **36**, 1162–1166 (2008).

17. Gros, M. *et al.* Veterinary pharmaceuticals and antibiotics in manure and slurry and their fate in amended agricultural soils: Findings from an experimental field site (Baix Empordà, NE Catalonia). *Sci. Total Environ.* **654**, 1337–1349 (2019).

18. Jansen, L. J. M. *et al.* A new extraction procedure to abate the burden of non-extractable antibiotic residues in manure. *Chemosphere* **224**, 544–553 (2019).

19. Wu, Y., Williams, M., Smith, L., Chen, D. & Kookana, R. Dissipation of sulfamethoxazole and trimethoprim antibiotics from manure-amended soils. *J. Environ. Sci. Heal. - Part B Pestic. Food Contam. Agric. Wastes* **47**, 240–249 (2012).

20. Kang, D. H. *et al.* Antibiotic uptake by vegetable crops from manure-applied soils. *J. Agric. Food Chem.* **61**, 9992–10001 (2013).

21. Montemurro, N., Postigo, C., Chirón, S., Barcelò, D. & Pérez, S. Analysis and fate of 14 relevant wastewater-derived organic pollutants in long-term exposed soil. *Anal. Bioanal. Chem.* **411**, 2687–2696 (2019).

22. Koba, O., Golovko, O., Kodešová, R., Fér, M. & Grabic, R. Antibiotics degradation in soil: A case of clindamycin, trimethoprim, sulfamethoxazole and their transformation products. *Environ. Pollut.* **220**, 1251–1263 (2017).

23. Qian, M. *et al.* Occurrence of trace elements and antibiotics in manure-based fertilizers from the Zhejiang Province of China. *Sci. Total Environ.* **559**, 174–181 (2016).

24. Zhou, L. J. *et al.* Simultaneous determination of human and veterinary antibiotics in various environmental matrices by rapid resolution liquid chromatography-electrospray ionization tandem mass spectrometry. *J. Chromatogr. A* **1244**, 123–138 (2012).

25. Van den Meersche, T. *et al.* Development, validation and application of an ultra high performance liquid chromatographic-tandem mass spectrometric method for the simultaneous detection and quantification of five different classes of veterinary antibiotics in swine manure. *J. Chromatogr. A* **1429**, 248–257 (2016).

26. Solliec, M. *et al.* Fractionation and analysis of veterinary antibiotics and their related degradation products in agricultural soils and drainage waters following swine manure amendment. *Sci. Total Environ.* **543**, 524–535 (2016).

27. Gómez-Granados, F., Codony, R., Granados, M. & Prat, M. D. Fluoroquinolones in soils: Assessment of extraction methods. *Int. J. Environ. Anal. Chem.* **91**, 1353–1366 (2011).

28. Golovko, O. *et al.* Development of fast and robust multiresidual LC-MS/MS method for determination of pharmaceuticals in soils. *Environ. Sci. Pollut. Res.* **23**, 14068–14077 (2016).

29. Martínez-Carballo, E., González-Barreiro, C., Scharf, S. & Gans, O. Environmental monitoring study of selected veterinary antibiotics in animal manure and soils in Austria. *Environ. Pollut.* **148**, 570–579 (2007).

30. Bianchi, S. R., Miyazawa, M., De Oliveira, E. L. & Pavan, M. A. Relationship between the mass of organic matter and carbon in soil. *Brazilian Arch. Biol. Technol.* **51**, 263–269 (2008).

31. Mitchell, P. J. & Simpson, M. J. High affinity sorption domains in soil are blocked by polar soil organic matter components. *Environ. Sci. Technol.* **47**, 412–419 (2013).

32. Svahn, O. & Björklund, E. Thermal stability assessment of antibiotics in moderate temperature and subcriticalwater using a pressurized dynamic flow-through system. *Int. J. Innov. Appl. Stud.* **11**, 872–880 (2015).

33. Kokoszka, K., Kobus, A. & Bajkacz, S. Optimization of a method for extraction and determination of residues of selected antimicrobials in soil and plant samples using HPLC-UV-MS/MS. *Int. J. Environ. Res. Public Health* **18**, 1–14 (2021).

34. Kokoszka, K., Wilk, J., Felis, E. & Bajkacz, S. Application of UHPLC-MS/MS method to study occurrence and fate of sulfonamide antibiotics and their transformation products in surface water in highly urbanized areas. *Chemosphere* **283**, 131189 (2021).
